# Elucidating the mechanism behind and investigating the efficacy of Traditional Chinese Medicine and Traditional Tibetan Medicine in combination with standard therapeutics in hepatocellular carcinoma and cholangiocarcinoma *in vitro*

**DOI:** 10.3389/fphar.2022.906468

**Published:** 2022-09-12

**Authors:** Huizhen Suo, Inga Hochnadel, Nataliia Petriv, Raimo Franke, Jennifer Schmidt, Nataliia Limanska, Alisa Tugai, Nils Jedicke, Mark Broenstrup, Michael P. Manns, Tetyana Yevsa

**Affiliations:** ^1^ Department of Gastroenterology, Hepatology and Endocrinology, Hannover Medical School, Hannover, Germany; ^2^ Department of Chemical Biology, Helmholtz Centre for Infection Research, Braunschweig, Germany; ^3^ Department of Microbiology, Virology and Biotechnology, Odesa I. I. Mechnykov National University, Odesa, Ukraine; ^4^ German Center for Infection Research, Braunschweig, Germany

**Keywords:** primary liver cancer, Huaier, Ershiwuwei Songshi Wan, Qiwei Honghua Shusheng Wan, senescence, apoptosis

## Abstract

In this study, we investigated compounds of plant and mushroom origin belonging to Traditional Chinese Medicine (TCM) and to Traditional Tibetan Medicine (TTM): a sandy beige mushroom *Trametes robiniophila* Murr, commonly known as Huaier/TCM as well as Ershiwuwei Songshi Wan and Qiwei Honghua Shusheng Wan, which both belong to TTM. We aimed to study the efficacy of TTM and TCM in hepatocellular carcinoma (HCC) and cholangiocarcinoma (CCA) *in vitro*. TCM and TTM were tested either as a monotherapy, or in combination with standard therapeutics: sorafenib for HCC treatment and gemcitabine for CCA. We also discovered a protective mechanism behind the most successful therapeutic combinations. The results demonstrated that TCM and TTM inhibited the proliferation of cancer cells in a time- and dose-dependent manner. The results were compared to classical chemotherapeutics currently used in the clinic: sorafenib for HCC and gemcitabine for CCA. In HCC settings, a combination of Huaier (16 mg/ml) with half of the human plasma concentration of sorafenib, Qiwei Honghua Shusheng Wan (1 mg/ml) monotherapy as well as its combination with half or even a quarter dose of the human plasma concentration of sorafenib represented the most efficient treatments, inhibiting the growth of HCC cells more effectively than the standard therapy. The inhibitory mechanism relied on a strong induction of apoptosis. In CCA settings, Ershiwuwei Songshi Wan and Qiwei Honghua Shusheng Wan as monotherapies or in combination with very low doses of gemcitabine inhibited the growth of CCA cells more efficiently than the standard therapy. Importantly, Ershiwuwei Songshi Wan at the 8 and 16 mg/ml concentrations and Qiwei Honghua Shusheng Wan at the 4 mg/ml concentration were efficacious with gemcitabine applied at massively reduced concentrations. The protective mechanism in CCA relied on a strong induction of early and late apoptosis. Cellular senescence and necroptosis were not associated with protection against HCC/CCA. Combination therapy with TCM or TTM allowed for a dose reduction of standard chemotherapeutics. This is especially important as both chemotherapeutic drugs show strong side effects in patients. The reduction of chemotherapeutics and the synergistic effect observed while applying them in combination with TCM and TTM has strong perspectives for the clinic and patients suffering from HCC and CCA.

## Graphical Abstract

Primary murine HCC and CCA cell lines with known genotypes were used to test the efficacy of TCM (Huaier) and TTM (Ershiwuwei Songshi Wan and Qiwei Honghua Shusheng Wan) applied as monotherapy and in combination with standard chemotherapeutics, sorafenib for HCC and gemcitabine for CCA. The results showed that TCM and TTM and their combination with standard chemotherapeutics were more efficient than the clinical doses of standard chemotherapeutics alone (13.8 μM sorafenib and 50 μM gemcitabine, respectively). In particular, monotherapy with TTM Qiwei Honghua Shusheng Wan and its combination with a half or even quarter dose of sorafenib (6.9 or 3.45 μM), as well as combination of TCM Huaier and 6.9 μM sorafenib strongly inhibited the growth of HCC cells. The HCC cell inhibition relied on the induction of late apoptosis. For CCA, monotherapy with both tested TTM compounds, Ershiwuwei Songshi Wan and Qiwei Honghua Shusheng Wan and their combination with reduced doses of gemcitabine (0.025, 0.05 or 0.1 μM) strongly inhibited CCA cell growth. The CCA cell inhibition relied on the induction of early- and late apoptosis. TCM Huaier was inefficient in CCA. Cellular senescence and necroptosis, induced by the compounds, did not correlate with the protection against HCC and CCA in *in vitro* studies.
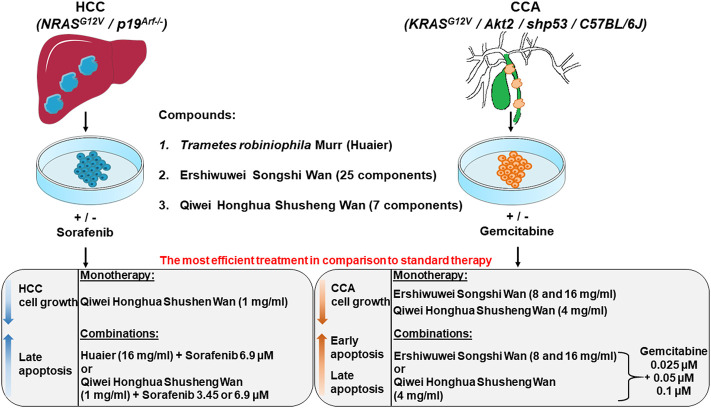

## 1 Introduction

Primary liver cancer (PLC) ranks sixth in cancer incidence and third in cancer-related mortality worldwide (Mak and Kramvis, 2021; Sung et al., 2021). Hepatocellular carcinoma (HCC) and intrahepatic cholangiocarcinoma (iCCA) are the most common types of PLC (Mejia and Pasko, 2020; McGlynn et al., 2021). Liver stem cells and mature hepatocytes genetically predisposed or affected by cirrhosis resulting from viral hepatitis infections, increased alcohol consumption, aflatoxin or other unfavourable factors, give rise to HCC (Llovet et al., 2021). CCA arises at bile or hepatic ducts or their junctions, and such types of malignancies often emerge in the non-cirrhotic liver (Banales et al., 2020) (Lee and Lee, 2017).

Treatment options for advanced HCC and CCA remain extremely limited. Moreover, anticancer therapy is often exhaustive for the patients due to a high toxicity of the standard chemotherapeutics, e.g., sorafenib for HCC (Raoul et al., 2019) and gemcitabine for CCA (Hryciuk et al., 2018). Combination therapy, which would allow a decrease in the doses of chemotherapeutics, would be of advantage in treatment of liver cancer (Walcher et al., 2020).

Traditional Chinese Medicine (TCM) and Traditional Tibetan Medicine (TTM) has a long and successful history for treating cancer in China (Luo et al., 2015; Li et al., 2018), and to date, the Guidelines of Diagnosis and Therapy in Oncology using TCM and TTM was generated with international standards consistent with modern clinical practice (Li et al., 2018; Tang et al., 2020). In this study, we concentrated on a TCM representative, a sandy beige mushroom *Trametes robiniophila* Murr, known in Chinese as “槐耳” or “Huaier”. Huaier grows on *Sophora japonica L.* tree trucks and belongs to *Hymenomycetes*, *Basidiomycotina* (Anithworth et al., 1973; Rogerson, 1974; Li et al., 2007; Sun et al., 2013). Huaier is commonly used in China for cancer complementary therapy including HCC treatment (Shan et al., 2017; Zhao et al., 2017; Pan et al., 2019; Li et al., 2020a; Tang et al., 2020). For TTM, we focused on: 1) a 25 component representative known in Chinese as “二十五味松石丸” or “Ershiwuwei Songshi Wan”; and 2) a 7 component representative known in Chinese as “七味红花殊胜丸” or “Qiwei Honghua Shusheng Wan”. Both TTM representatives include a rich variety of plants and minerals, among them are *Aristolochia debilis* Siebold & Zucc.*, Meconopsis cabrica* Vig*, Terminalia chebula* Retz.*, Bambusa testilis* McClure, and others, which all are depicted in Supplementary Figures S1, S2 and liquid chromatography mass spectrometry (LC-MS/MS) profiles for the compounds are provided in Supplementary Figure S3.

Importantly, Ershiwuwei Songshi Wan and Qiwei Honghua Shusheng Wan have been in use for the treatment of liver injuries such as hepatitis, cholecystitis, cirrhosis and other diseases (Qi et al., 2000; Yang, 2000; Ci et al., 2018; Ma et al., 2020; Yan Xi et al., 2020). The long history of these compounds in TCM and TTM for the treatment of liver diseases encouraged us to evaluate the potential of Huaier [designated later on in this study as compound 1 (C1)], Ershiwuwei Songshi Wan (designated as C2) and Qiwei Honghua Shusheng Wan (designated as C3) in two types of PLC, HCC and CCA, in an *in vitro* setting.

Importantly, along with monotherapeutic regimes using C1, C2 and C3 alone, we also tested their combinations with standard chemotherapeutics, sorafenib for HCC and gemcitabine for CCA. Standard chemotherapeutics applied at their plasma concentrations (defined for human) as well as carriers served as controls. We searched for synergistic effects and inhibition mechanisms while investigating the efficacy of TCM and TTM in combination with standard chemotherapeutics. We also explored the opportunity to reduce the dose of chemotherapeutics and evaluated the synergistic effects with TCM and TTM in HCC and CCA.

## 2 Experimental procedures

### 2.1 Establishment and cultivation of hepatocellular carcinoma and cholangiocarcinoma cell lines

HCC and CCA cell lines used in this study were isolated from murine PLC. HCC was induced using intrahepatic overexpression of oncogenic *NRAS* ^*G12V*^ stably delivered *via* hydrodynamic tail vein injection into the liver of mice with a tumor suppressor *Arf* (p19^Arf−/−^ mice) deficiency (Carlson et al., 2005; Kang et al., 2011). CCA was induced *via* stable intrahepatic integration of a transposon mixture encoding: *KRAS* ^*G12V*^ and *Akt2* oncogenes as well as a specific aberration of p53 using short hairpins *(shp53)*, stably delivered into hepatocytes of C57BL/6J wild type mice using an electroporation technique (Gurlevik et al., 2013; Gurlevik et al., 2016). Both cell lines were cultivated in complete Dulbecco’s Modified Eagle’s Medium (DMEM; Gibco, United States), which was supplemented with 10% Fetal Bovine Serum (FBS; Gibco, United States), 5% penicillin/streptomycin/glutamine (Gibco, United States) and 5% Minimum Essential Medium Non-Essential Amino Acids (MEM NEAA, Gibco, United States). Cell lines were incubated at 37°C in a humidified incubator supplied with 5% CO_2_.

### 2.2 Preparation of Traditional Chinese Medicine and Traditional Tibetan Medicine

C1 [*T. robiniophila* Murr (Huaier), TCM] is commercially available and was purchased from the company Qidong Gaitianli Pharmaceuical Co., Ltd., Jiangsu, China. The compound is made of granules based on 100% *T. robiniophila* Murr*,* a sandy beige mushroom. C2 (Ershiwuwei Songshi Wan, TTM) and C3 (Qiwei Honghua Shusheng Wan, TTM) are commercially available and were both purchased from the company Tibet Ganlu Tibetan Medicine Co., Ltd., Lhasa, China. All names of plants used in the polyherbal formulations were verified using the following databases: http://mpns.kew.org/mpns-portal/, http://www.plantsoftheworldonline.org/ and www.theplantlist.org. The content and proportions of plants in polyherbal formulations are presented in Supplementary Figure S1 for C2, Supplementary Figure S2 for C3 as well as Supplementary Table S1.

For the experiments, C1, C2 and C3 were processed to fine powder using a mortar. The fine powders were completely dissolved at a concentration of 100 mg/ml in DMEM in a 37°C water bath for 15 min. The obtained extracts were filtered through 0.22 μm membrane filters (Fischerbrand, PTFE). The resulting stock solutions were kept frozen at −20°C.

### 2.3 Treatment of hepatocellular carcinoma and cholangiocarcinoma cell lines with Traditional Chinese Medicine and Traditional Tibetan Medicine

**FIGURE 1:**
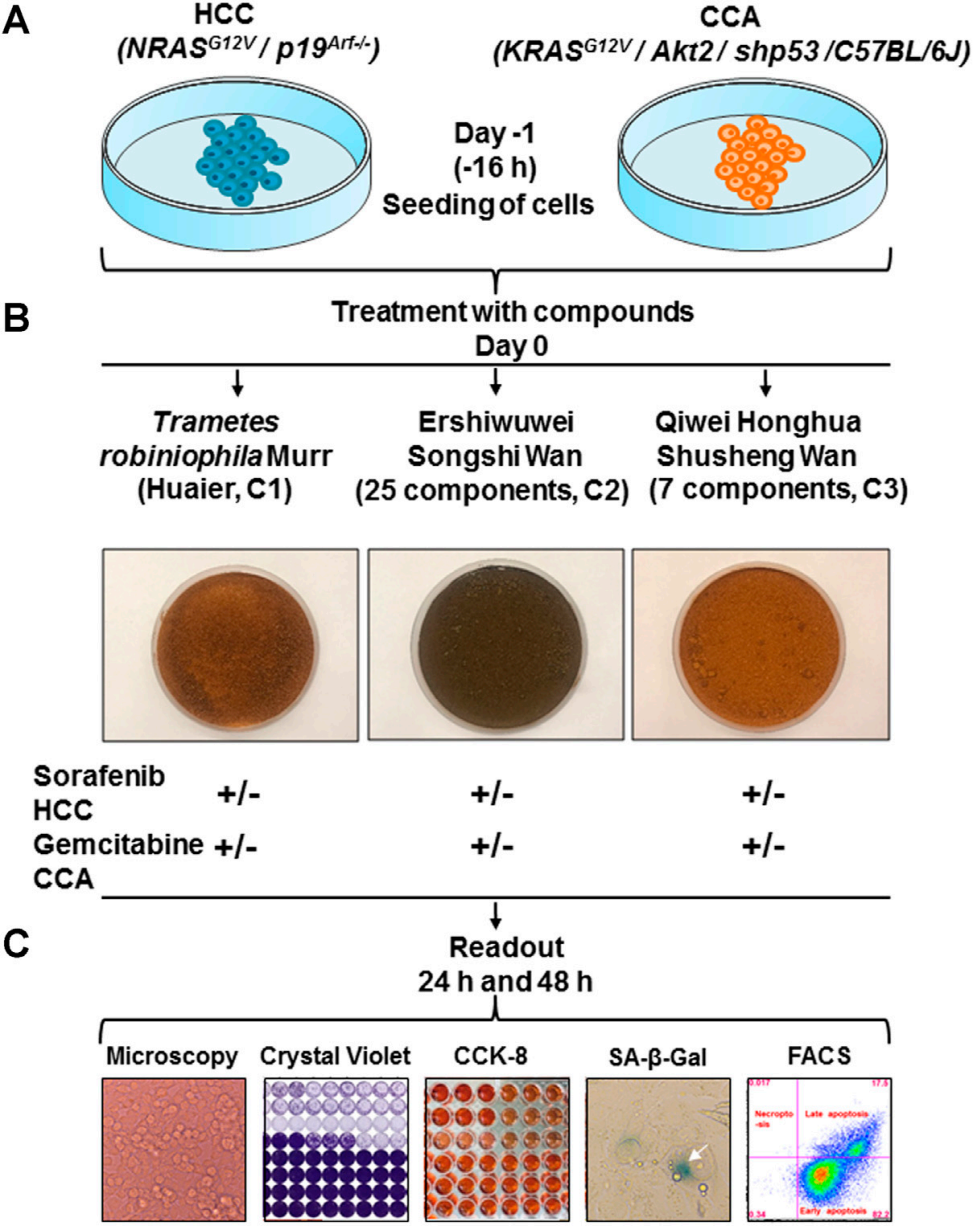
Experimental layout to study TCM, TTM, and the mechanism of their action *in vitro* using HCC and CCA cell lines. **(A)** Cells were seeded on day—1; **(B)** on day 0 compounds C1, C2 and C3 were added at different concentrations as a monotherapy or in combination with standard chemotherapeutics; **(C)** after 24 and 48 h different readouts were performed and comprised bright field microscopy, CVSA, CCK-8 and SA-β-Gal assays as well as FACS analysis.

HCC and CCA cells were seeded in 96-well plates (Corning, Inc. United States) at a density of 1 × 10^4^ cells per well (see experimental layout in Figures 1A–C). TCM (C1) and TTM (C2 or C3), as well as the standard therapeutics (sorafenib for HCC and gemcitabine for CCA), were added to the wells 16 h later in triplicates.

For cell culture experiments, the stock solutions of C1 were diluted with DMEM to obtain the final concentrations of 4, 8 and 16 mg/ml for both, HCC and CCA. C2 was used at the final concentrations of 1, 2 and 4 mg/ml for HCC and 4, 8 and 16 mg/ml for CCA. C3 was used at the final concentrations 0.25, 0.5 and 1 mg/ml for HCC and 1, 2 and 4 mg/ml for CCA (Supplementary Table S2). Sorafenib was used at concentrations 3.45, 6.9, and 13.8 μM, where 13.8 µM represents the human plasma concentration of sorafenib, as reported (Fucile et al., 2015). Gemcitabine concentrations were used as follows: 0.025, 0.05, 0.1 and 50 μM, where 50 μM represents human plasma concentration, as reported (Fujiwara et al., 2015) (Supplementary Table S2). Compounds were tested on the cell lines alone and/or in combination with standard chemotherapeutics: sorafenib for HCC and gemcitabine for CCA. Chemotherapeutics as well as carrier served as controls.

Different readouts were conducted at 24 and 48 h post-incubation with TCM, TTM and standard therapeutics (Figures 1A–C).

### 2.4 Crystal violet staining assay

Crystal violet staining assay (CVSA) was performed as previously described (Rudalska et al., 2014; Śliwka et al., 2016; Pylypchuk et al., 2022). Briefly, cells were first washed with 100 μl 1 × phosphate buffered saline (PBS) and fixed using 100 μl of 4% paraformaldehyde. Thereafter, cells were stained with 100 μl of 0.5% crystal violet (Sigma-Aldrich Corp., St. Louis, MO, United States) in 30% ethanol. Finally, cells were washed in tap water and dried overnight. The microphotographs were taken using an ImmunoSpot^®^ S6 ULTIMATE Analyzer (Cellular Technology Limited, United States). The obtained microphotographs were analyzed using ImageJ software (https://imagej.nih.gov/ij/index.html).

### 2.5 Cell proliferation assay/cell counting kit-8

Cell counting kit-8 (CCK-8) assay (Sigma-Aldrich, United States) was used to determine cell proliferation and was performed using a manufacturer protocol, as recently described (Pylypchuk et al., 2022). Cell proliferation was checked at two time points, 24 and 48 h post-incubation with TCM and TTM. 10 μl CCK-8 solution was added to each well, incubated for 2 hours, measured at an optical density (OD) 450 nm using an Infinite 200 PRO Nano Quant Tecan Microplate Reader (TECAN, CH-8708 Mannedorf, Switzerland) and analyzed using i-controlTM software, as described (Zhou et al., 2018; Pylypchuk et al., 2022).

### 2.6 Senescence β-galactosidase assay

Senescence β-galactosidase assay (SA-β-Gal) was performed as previously described (Kang et al., 2011; Cahu and Sola, 2013; Eggert et al., 2016; Pylypchuk et al., 2022). Briefly, cells were first washed with PBS (pH 7.2–7.4) and fixed with 2% formaldehyde and 0.2% glutaraldehyde solution in PBS (pH 7.2–7.4). Thereafter, cells were incubated at 37°C in the staining solution (potassium ferrocyanide, potassium ferricyanide and X-galactose in PBS supplemented with 1 mM MgCl_2_, pH 6.0, Sigma-Aldrich, United States). After the development of blue stain in experimental groups, the staining was stopped. Five high power field photos from each well were taken (objective ×40) using the Nikon microscope Eclipse Ti2 (Nikon, Japan).

### 2.7 Analysis of senescence, early/late apoptosis and necroptosis using flow cytometry

Cellular senescence response, early and late apoptosis, as well as necroptosis were detected by flow cytometry (FACS) as previously described by (Cahu and Sola, 2013; Bushnell, 2015; Pylypchuk et al., 2022). First, a detection of cellular senescence was performed by staining with 5-dodecanoylaminofluorescein di-β-D-galactopyranoside (C_12_FDG) (Thermo Fisher Scientific, D2893, Germany) (Cahu and Sola, 2013). Briefly, cells were initially treated with 100 nM bafilomycin A1 (Merck, 196000, United States) for 1 h to accomplish lysosomal alkalization, followed by an incubation with C_12_FDG (20 mM) in complete DMEM (Gibco, United States) medium for 2 h at 37°C in a humidified incubator supplied with 5% CO_2_. Then cells were washed with pre-warmed PBS (pH 7.2–7.4), trypsinized and the resulting cell suspension was transferred into FACS tubes. Thereafter, staining with Annexin V-Phycoerythrin (PE) and 7-Amino-Actinomycin (7-AAD) was performed to detect early and late apoptosis, as well as necroptosis, as described (Bushnell, 2015). Briefly, 5 μl of Annexin V-PE (Biolegend^®^, United States), 5 μl of 7AAD (Biolegend^®^, United States) and 400 μl of Annexin V binding buffer (Biolegend^®^, United States) were added to each tube and cell suspensions were stained on ice for 20 min, protected from light. After the incubation, the samples were pooled from three independent experiments and acquired using a flow cytometer (BD™ LSR II, San Jose, CA, United States). FACS analysis was performed using the FlowJo 9.9.6 software (BD™, United States).

### 2.8 Liquid chromatography mass spectrometry analysis

Acetonitrile ultra LC/MS grade, water ultra LC/MS grade, and formic acid ultra LC/MS grade were obtained from Fisher Scientific. For the LC-MS/MS-analysis, the powder of C1, C2, or C3 was dissolved at a concentration of 100 mg/ml in sterile MQ water and incubated at 37°C (water bath) for 15 min. The obtained extracts were filtered through a 0.45 μM membrane filter (Fischerbrand, PTFE). A blank sample was prepared using the same MQ water and filter procedure. For each sample, 2 μl of the stock solution was analyzed by reversed phase ultrahigh-performance liquid chromatography coupled to trapped ion mobility quadrupole time-of-flight mass spectrometry. Each sample was analyzed in triplicate. The samples were separated using ultra high-performance liquid chromatography, performed on a Dionex Ultimate 3000 UPLC system (Thermo Fisher Scientific, Waltham, MA, United States) using a 150 by 2.1 mm Kinetex C18 column with 1.7 μm particle size (Phenomenex, Aschaffenburg, Germany) column with a flow rate of 300 μl/min. Gradient elution with water with 0.1% (vol/vol) formic acid as eluent A and acetonitrile with 0.1% (vol/vol) formic acid as eluent B was run as follows: 1% B for t = 0 min to t = 2 min, linear gradient from 1% B to 100% B from t = 2 min to t = 20 min, hold 100% B until t = 25 min, and linear gradient from 100% B to 1% B from t = 25 min to t = 30 min.

The samples were analyzed by positive mode electrospray ionization trapped ion mobility quadrupole time-of-flight mass spectrometry on a timsTOF Pro instrument (Bruker, Bremen, Germany) in data-dependent MS2 mode (tims on, 20–1000 Da). ESI source parameters were 10 L/min drying gas at 220°C, 4500 V capillary voltage and 2.2 bar nebulizer pressure. Base peak chromatograms were generated with Bruker Compass DataAnalysis 5.3. Peak tables were generated using the Bruker Software Metaboscape 2022. The peak tables were annotated using the smart formula algorithm to determine the most probable sum formulas. The mean of the blank runs (triplicate injections) was subtracted from the mean of the sample runs (triplicate injections). Only features with significantly different intensities above the blank (Welch’s t-test, *p* < 0.05) were kept.

### 2.9 Statistical analysis

The tests with all compounds were performed in triplicates. If not stated otherwise, the unpaired Student’s *t*-test was used for statistical analyses to calculate significant differences among experimental and control groups. Unless stated otherwise, data are depicted as mean ± standard error of the mean (SEM) with *p* < 0.05 considered as statistically significant. Significance levels were represented as *p < 0.05, **p < 0.01, ***p < 0.001, and ****p < 0.0001.

## 3 Results

We first established HCC and CCA cells lines from primary murine HCC and CCA, both with known genotype (see an experimental setup in Figure 1A). The obtained HCC and CCA cells were seeded 16 h prior to the addition of compounds (Figure 1A). Thereafter, the freshly prepared DMEM extracts from all three compounds (C1, C2, C3) were added in triplicates at different concentrations as 1) monotherapy, or as 2) combination with standard chemotherapeutics (sorafenib for HCC and gemcitabine for CCA) (Figure 1B; Supplementary Table S2). After 24 and 48 h of incubation, the diverse readouts such as: microscopy, CVSA, CCK-8 and SA-β-Gal assays, as well as FACS were performed to assess the inhibitory property of therapeutic regimes and to define a main mechanism beyond the inhibition (Figure 1C).

In addition, we performed a detailed characterization of C1, C2 (Supplementary Figure S1) and C3 (Supplementary Figure S2) using LC-MS/MS as depicted in the Supplementary Figure S3. In order to characterize the constituents of C1, C2 and C3 water extracts of the samples were analyzed by reversed phase ultrahigh-performance liquid chromatography coupled to trapped ion mobility quadrupole time-of-flight mass spectrometry. All extracts were featured by highly complex compositions (Supplementary Figure S3). They mainly contained hydrophilic to mid-polar compounds, with C2 containing most of the mid-polar compounds. In order to get a first overview on individual metabolites, we performed a feature extraction to generate peak tables for each sample. After blank subtraction, we were able to detect 2330 features in C1, 3678 in C2 and 3377 features in C3. Using lockmass-calibrated high resolution masses and the smartFormula algorithm of Metaboscape 2022, we determined the most probable sum formulas where possible (Supplementary Table S3).

### 3.1 Hepatocellular carcinoma

#### 3.1.1 C1 and C3 alone and in combination with sorafenib demonstrated an inhibitory effect on hepatocellular carcinoma cell growth as detected by crystal violet staining assay

**FIGURE 2:**
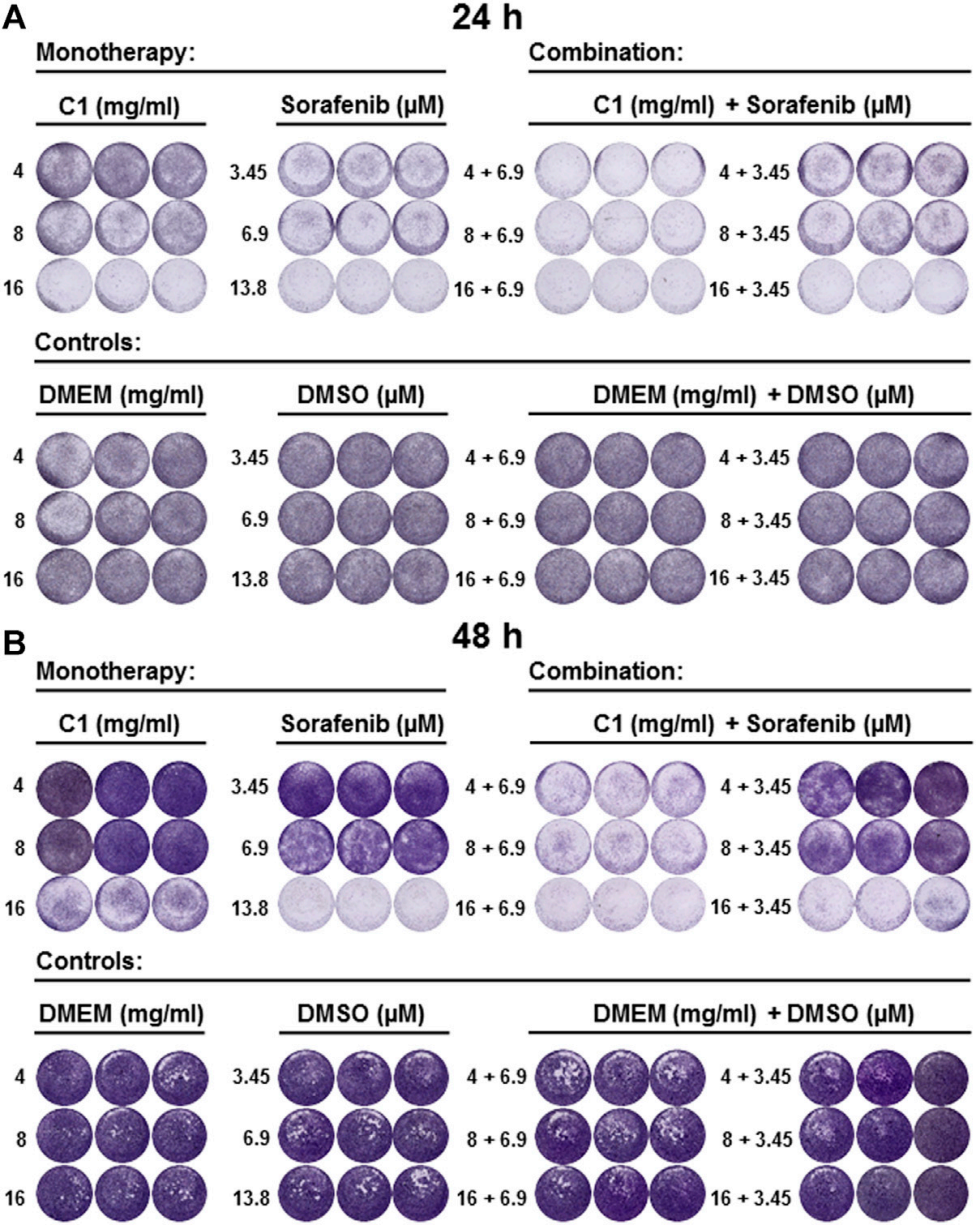
C1 and its combination with sorafenib inhibited the growth of HCC in a dose-dependent manner. CVSA was performed in HCC cells after the treatment with C1 alone at different concentrations (4, 8 and 16 mg/ml) or in combination with sorafenib at different concentrations (3.45, 6.9 and 13.8 μM). CVSA readouts were performed **(A)** 24 h and **(B)** 48 h post-incubation. For a direct comparison to the standard therapy in the clinic, the sorafenib dose of 13.8 μM [human plasma concentration, as reported for the clinic (Fucile et al., 2015)], was used as a positive control.

We first performed CVSA to test the inhibitory capacity of C1 in HCC cells (Figure 2A) and analyzed the results by using ImageJ (Supplementary Figure S4; Supplementary Table S4). Monotherapy with C1 inhibited HCC cell line growth already after 24 h of incubation in comparison to untreated controls (Figure 2A; Supplementary Figure S4A; Supplementary Table S4). Standard chemotherapeutic sorafenib also inhibited the HCC growth, as expected (Figure 2A; Supplementary Figure S4A; Supplementary Table S4). The effect was dose-dependent in C1 (Figure 2A; Supplementary Figure S4A; Supplementary Table S4). Importantly, the concentration 16 mg/ml of C1 showed the highest inhibitory effect that was significant and comparable to the human plasma concentration of standard chemotherapeutic sorafenib (13.8 μM) as shown in (Figure 2A; Supplementary Figure S4A; Supplementary Table S4). Strikingly, a combination therapy with C1 in all the tested concentrations (4, 8, 16 mg/ml) using sorafenib at half of the plasma concentration (6.9 μM) resulted in almost 100% inhibition of HCC growth (Figure 2A; Supplementary Figure S4A; Supplementary Table S4). Further, reduction of sorafenib (3.45 μM) was efficient only in combination with the highest dose, 16 mg/ml, of C1 (Figure 2A; Supplementary Figure S4A; Supplementary Table S4). The CVSA results were further analysed after 48 h of incubation (Figure 2B; Supplementary Figure S4B; Supplementary Table S4) and confirmed the most efficacious treatment in combination of 1) C1 (4, 8, 16 mg/ml) and 6.9 µM sorafenib or 2) C1 (16 mg/ml) and 3.45 μM sorafenib (Figure 2B; Supplementary Figure S2B; Supplementary Table S4). C1 16 mg/ml and 6.9 μM sorafenib showed similar efficacy as sorafenib alone at the 13.8 μM concentration (Figure 2B; Supplementary Figure S4B; Supplementary Table S4). Inhibition of HCC growth was not detected in the negative controls, DMEM and DMSO, used as carriers for C1 and sorafenib, respectively (Figures 2A,B; Supplementary Figure S4A,B; Supplementary Table S4).

We further tested CVSA, applying C2, a 25-component drug (Supplementary Figures S5, S6) on a HCC cell line. We performed treatment with C2 as monotherapy and in combination with sorafenib and analysed CVSA 24 and 48 h later (Supplementary Figures S5A, S6A for 24 h, Supplementary Figures S5B, S6B for 48 h and Supplementary Table S4). Interestingly, C2 alone did not inhibit the HCC cell line. However, it exhibited a synergistic effect with sorafenib: the addition of 1, 2 or 4 mg/ml of C2 to 6.9 μM of sorafenib significantly increased the efficacy of the standard chemotherapeutic drug (Supplementary Figures S5A,B, S6A,B; Supplementary Table S4). Less efficient but still a significant effect of C2 with 3.45 µM of sorafenib also demonstrated an inhibitory effect on HCC, however it was much less than the one of standard therapy (Supplementary Figures S5A,B, S6A,B; Supplementary Table S4). In general, at 24 h the formulations based on C2 showed similar efficacy as the human plasma concentration of sorafenib (13.8 µM) but the effect diminished 48 h post treatment as demonstrated in Supplementary Figures S5, S6.

We further tested C3, a 7-component drug (Supplementary Figure S2) in HCC using mono- and combination therapy approaches and then performing a CVSA assay and statistical analysis using ImageJ (Supplementary Figures S7A,B, S8A,B). C3 strongly inhibited HCC cell line growth when applied at the highest concentration (1 mg/ml) (Supplementary Figures S7A,B, S8A,B; Supplementary Table S4). C3 (1 mg/ml) alone or a combination of C3 (1 mg/ml) with sorafenib at a concentration of 6.9 μM or 3.45 µM was even more efficient than sorafenib (13.8 μM) monotherapy at 48 h post-incubation (Supplementary Figure S7B, S8B; Supplementary Table S4).

In summary, based on CVSA data, the most efficient treatments, which were superior to the plasma concentration of sorafenib (13.8 μM), were as follows: 1) combination of 16 mg/ml C1 with 6.9 μM sorafenib; 2) monotherapy with 1 mg/ml C3 and 3) combination of 1 mg/ml C3 with 6.9 or 3.45 μM sorafenib.

#### 3.1.2 Combination of C1, C2 and C3 with 6.9 μM sorafenib demonstrated a strong inhibitory effect on the hepatocellular carcinoma cell line in cell counting kit-8 analysis

**FIGURE 3:**
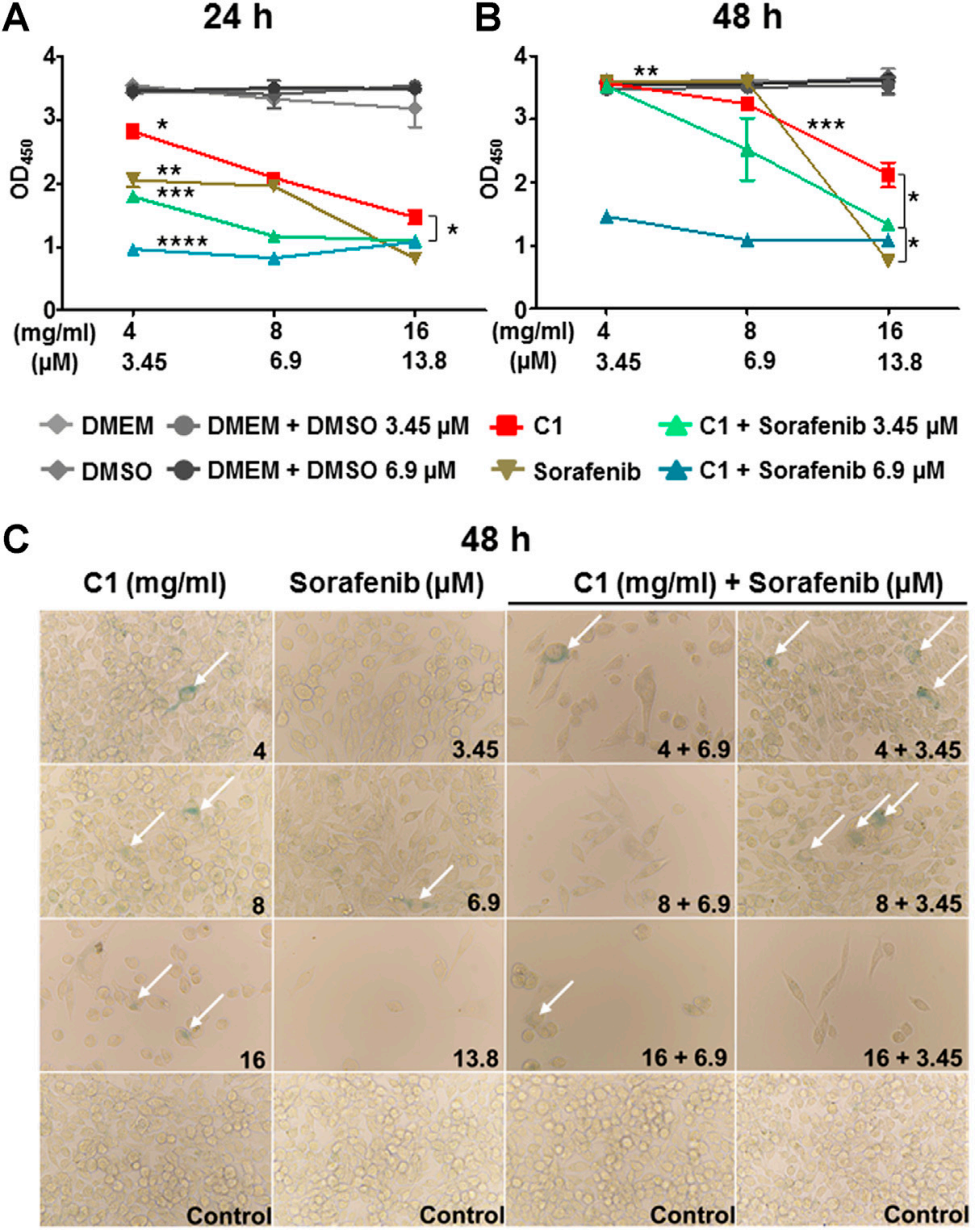
C1 and its combination with sorafenib demonstrated inhibitory effects on HCC cell line in CCK-8 analysis and induced cellular senescence. HCC cells were treated with C1 and its combination with sorafenib and **(A)** 24 h and **(B)** 48 h after incubation cells were subjected to CCK-8 analysis at OD_450._ Shown are mean ± SEM with *p < 0.05, **p < 0.01, ***p < 0.001, and ****p < 0.0001. **(C)** SA-β-Gal assay was performed 48 h post-incubation in C1-treated group. Shown are representative bright field microscopy pictures (objective ×40). Senescent (blue) cells are depicted with white arrows.

We further performed the CCK-8 assay to quantify the inhibitory capacity of applied compounds on HCC cell proliferation. We observed a dose-dependent inhibiting effect in C1 alone already at 24 h post-incubation (Figure 3A). Inhibition of cell proliferation with 16 mg/ml C1 was almost comparable with the inhibition induced by 13.8 µM plasma concentration of sorafenib, which also correlated with the CVSA data (Figure 2A; Supplementary Figure S4A). However, the most efficient inhibition of HCC, that was superior or similar to plasma concentration of sorafenib, was detected when C1 was applied at concentration of 16 mg/ml in combination with 6.9 μM of sorafenib, as verified at both tested time points, 24 and 48 h (Figures 3A,B, respectively). The data correlated with CVSA data analysis (Supplementary Figures S4A,B).

CCK-8 analysis confirmed the results of CVSA for compounds C2 and C3 (Supplementary Figures S9A,B for C2 and Supplementary Figures S9C,D for C3, respectively). Thus, at both tested time points, the most prominent results of cell growth inhibition were detected for the combinations of C2 with 6.9 μM sorafenib (Supplementary Figures S9A,B). Although not significant, a clear inhibitory effect of 1 mg/ml C3 as a monotherapy as well as of 1 mg/ml C3 with 6.9 or 3.45 µM of sorafenib could be seen at both time points and was more efficient or comparable to the effect of plasma concentration of sorafenib (Supplementary Figures S9C,D).

#### 3.1.3 Combination of C1 and C3 with sorafenib induced senescence in hepatocellular carcinoma cell line

We aimed further to test, whether compounds alone or in combination with sorafenib might result in cellular senescence induction and performed a SA-β-Gal staining in the HCC cell line after the treatments. Interestingly, the level of detected senescence in C1-treated wells was comparable with that induced by the multikinase inhibitor sorafenib in monotherapeutic regimes (Figure 3C). However, in combination, C1 (4 and 8 mg/ml) with 3.45 µM of sorafenib seemed to have a synergistic effect and more senescent cells (blue cells depicted with white arrows) were detected in those groups (Figure 3C). Similar results were obtained in C3- and to a lesser extent in C2-treated groups (Supplementary Figure S9E and data not shown). In general, cellular senescence did not seem to be the most important mechanism behind the inhibition, as it did not correlate much with the results from CVSA and CCK-8 assays in HCC settings and only few senescent cells could be detected upon treatments.

#### 3.1.4 C1, C2 and C3 in combination with lower doses of sorafenib induced late apoptosis and partially necroptosis in a dose-dependent manner in hepatocellular carcinoma cell line

**FIGURE 4:**
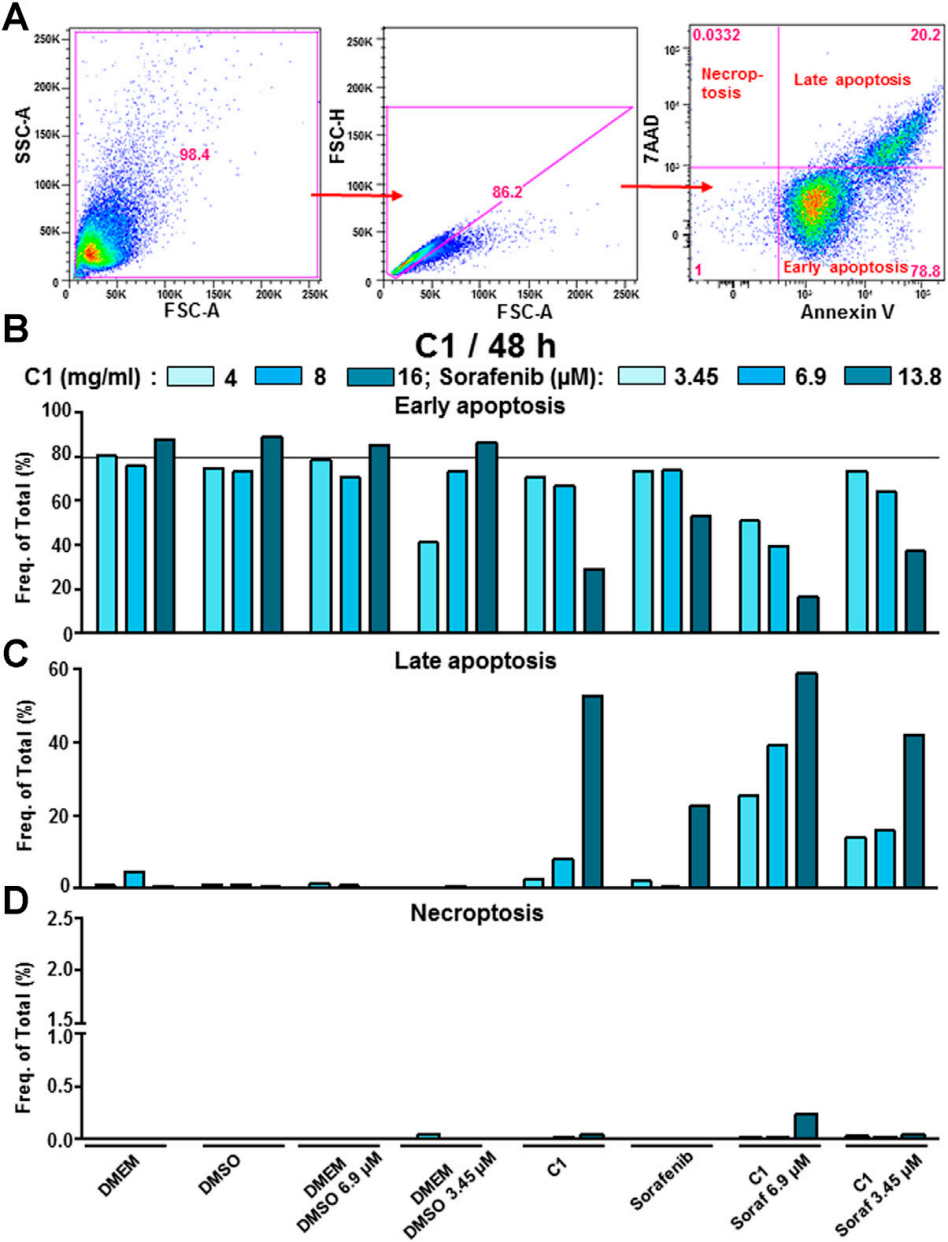
C1 and its combination with sorafenib induced mostly late apoptosis in HCC cells. **(A)** Gating strategy to analyze early, late apoptosis, and necroptosis. Cells were gated using forward- and side scatter characteristics while avoiding duplicates. The analysis of three different populations based on 7-AAD and Annexin V-PE stainings was performed. The upper left quadrant indicate necroptotic, the lower right and upper right quadrants indicate early and late apoptotic cells, respectively. The results of FACS analysis showing frequencies in percent of **(B)** early, **(C)** late apoptotic and **(D)** necroptotic cells are presented. DMSO and DMEM were used as carrier (negative controls) for sorafenib and C1, respectively. The grey line represents the values for the control group (DMEM).

Next, we performed FACS analysis to further define the mechanism of inhibition and to see whether the therapy might induce early or late apoptosis or necroptosis, or a combination thereof, using staining with Annexin V-PE and 7-AAD, as shown in gating strategy in (Figure 4A). We detected that sorafenib did not induce early, but late apoptosis and almost no necroptosis (Figures 4B–D). C1 alone worked similar to sorafenib and, surprisingly, induced even stronger late apoptosis and necroptosis in a dose-dependent manner, than sorafenib monotherapy (Figures 4B–D). A synergistic effect of C1 in all tested concentrations and 6.9 and 3.45 μM of sorafenib was clearly observed, whereas the combination C1 16 mg/ml and 6.9 μM sorafenib showed the strongest late apoptosis and necroptosis, that was superior to 13.8 plasma concentration of sorafenib (Figures 4C,D). Similarly to the results obtained for C1, treatment of HCC cell line with C2 and C3 mainly resulted in triggering late apoptosis, not early apoptosis phase (Supplementary Figures S10A–C for C2, and Supplementary Figures S11A–C for C3, respectively). C2 monotherapy in its highest concentration was less efficient in induction of late apoptosis, than sorafenib 13.8 µM plasma concentration, that correlated with CVSA data (Supplementary Figures S5, S6, S10B). However, combinations of C2 applied at its highest concentration with either 6.9 or 3.45 µM sorafenib increased the induction of late apoptosis, in comparison to 13.8 µM sorafenib monotherapy (Supplementary Figure S10B). In contrast to C2 and in line to C1, C3 monotherapy showed higher rate of late apoptosis in comparison to sorafenib monotherapy (Supplementary Figure S11B). Combination of C3 1 mg/ml and sorafenib 6.9 or 3.45 µM resulted in synergistic effect and induced late apoptosis more efficiently, than sorafenib plasma concentration alone (Supplementary Figure S11B). Interestingly, C2 alone or in combination with 3.45 μM sorafenib resulted in cells entering necroptosis (Supplementary Figure S10C), whereas C3 did not or barely induced necroptosis (Supplementary Figure S11C). Importantly, both C1 and C3 applied as monotherapy at the highest concentration induced stronger late apoptosis than sorafenib plasma concentration (13.8 µM), whereas C2 did not demonstrate such efficacy (Figure 4C; Supplementary Figures S10B, S11B). Similarly to C1, combination of the highest concentrations of C2 or C3 with 6.9 or 3.45 µM sorafenib resulted in stronger late apoptosis, than the one induced by 13.8 µM plasma concentration sorafenib (Supplementary Figures S10B, S11B). Interestingly, C1 demonstrated the highest rate or late apoptosis in comparison to C2 and C3 (Figure 4C; Supplementary Figures S10B, S11B), however, the latter data correlated neither with CVSA nor with CCK-8 data for C1.

### 3.2 Cholangiocarcinoma

#### 3.2.1 C2 and C3 alone and in combinations with gemcitabine demonstrated an inhibitory effect on the cholangiocarcinoma cell line shown in crystal violet staining assay experiments

We next investigated C1, C2 and C3 in CVSA experiments using the other PLC type, the CCA cell line. Surprisingly, C1 monotherapy did not inhibit the growth of CCA cells in any of the tested concentrations nor in combinations with 0.05 or 0.1 µM standard therapy gemcitabine (Supplementary Figure S12 for 24 h, Supplementary Figure S13 for 48 h, Supplementary Figure S14 and Supplementary Table S4 for 24 and 48 h). The standard chemotherapeutic gemcitabine tested at four concentrations (0.025, 0.05, 0.1 and 50 μM), where 50 µM represents a human plasma concentration (Fujiwara et al., 2015), resulted in CCA cell line inhibition when higher concentrations were applied (0.05, 0.1 and 50 μM, Supplementary Figures S12–S14). None of the negative controls demonstrated any changes in cell growth.

**FIGURE 5:**
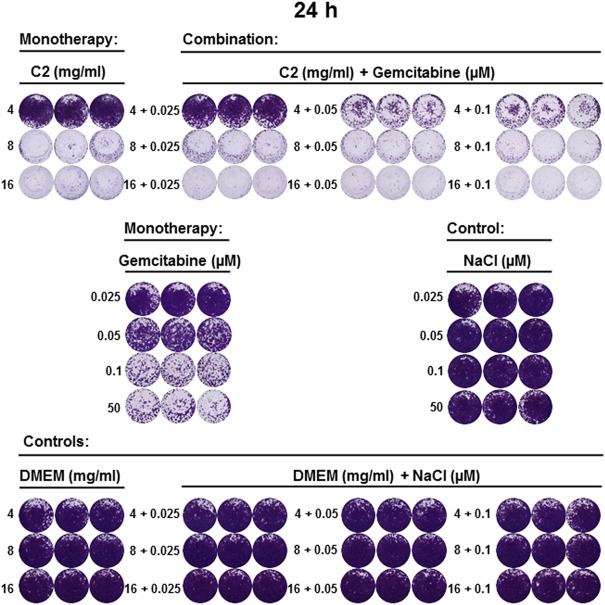
C2 alone and in combination with gemcitabine inhibited the growth of CCA cell line 24 h post-incubation. CVSA was performed to test the inhibitory capacity of C2 as a monotherapy and in combination with gemcitabine 24 h post-incubation. Carriers (DMEM for C2, NaCl for gemcitabine) were used as negative controls. Positive control (gemcitabine) was applied at different concentrations increasing from 0.025, 0.05, 0.1–50 μM [human plasma concentration (Fujiwara et al., 2015)].

**FIGURE 6:**
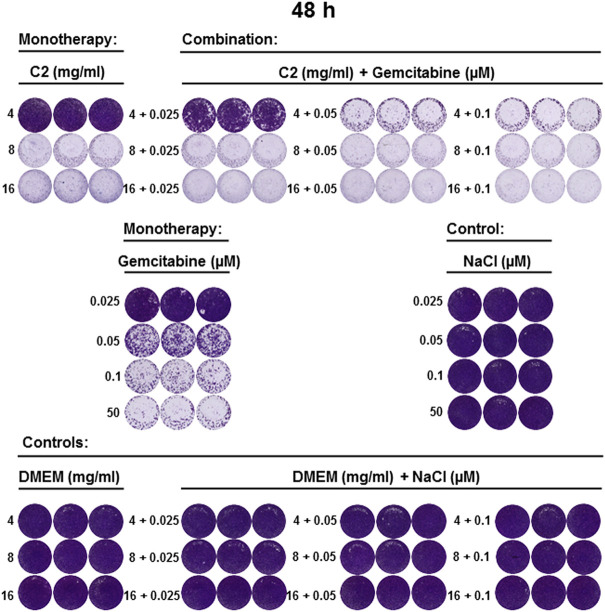
C2 alone and in combination with gemcitabine inhibited the growth of CCA cell line 48 h post-incubation. CVSA analysis performed as described in Figure 5 and analyzed 48 h after incubation.

**FIGURE 7:**
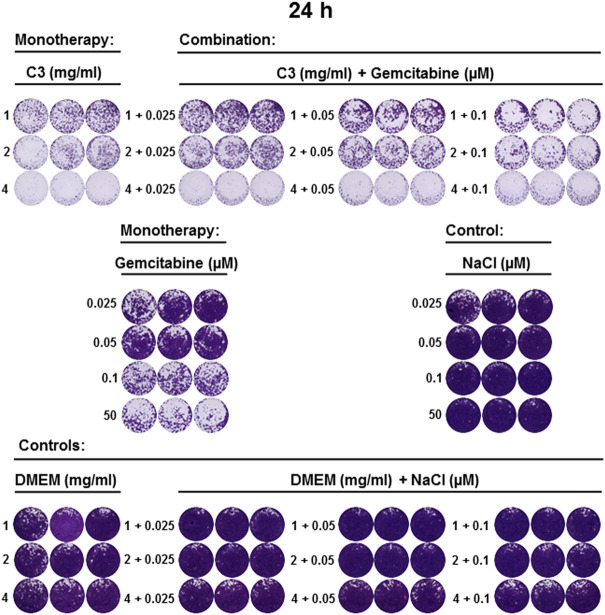
C3 alone and in combination with gemcitabine inhibited the growth of CCA cell line 24 h post-incubation. CVSA has been performed to test the inhibitory capacity of C3 in combinations with gemcitabine 24 h post-incubation. Positive and negative controls were included as described in Figure 5.

**FIGURE 8:**
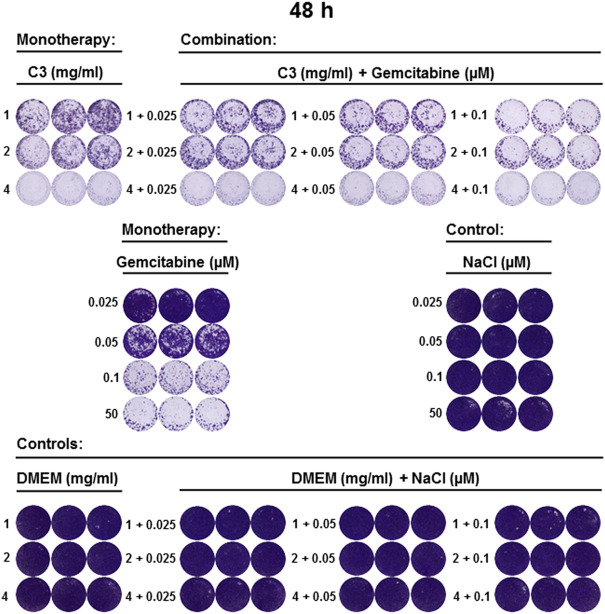
C3 alone and in combination with gemcitabine inhibited the growth of CCA cell line 48 h post-incubation. CVSA has been performed to test the inhibitory capacity of C3 in combinations with gemcitabine 48 h post-incubation. Positive and negative controls were included as described in Figure 5.

In contrast to C1, both C2 and C3 applied in monotherapeutic regime strongly inhibited CCA cell growth in a dose-dependent manner and combinations with gemcitabine further increased the efficacy of the formulations (Figures 5, 6; Supplementary Figure S15 for C2, Figures 7, 8; Supplementary Figure S16 for C3, Supplementary Table S4 for C2 and C3 24 and 48 h, respectively). Importantly, combinations of C2 with gemcitabine allowed for a decrease in the effective concentration of the standard chemotherapeutic from 50 μM (human plasma concentration) to 0.1, 0.05 or even to 0.025 μM (Figures 5, 6; Supplementary Figure S15). The latter was also true for C3-treated groups (Figures 7, 8; Supplementary Figure S16). Of note, monotherapies with C2 (at concentrations of 8 and 16 mg/ml) and C3 (at concentration of 4 mg/ml) were more effective than gemcitabine at the plasma concentration alone in inhibiting CCA, whereas combinations allowed for a reduction of both organic and chemotherapeutic drugs to achieve the same inhibitory effect (Figures 5–8).

In summary, C2 and C3 demonstrated high efficacy while inhibiting CCA growth, whereas C1 did not show such capacity.

#### 3.2.2 C1 and C3 induced senescence in the cholangiocarcinoma cell line

In line with HCC investigations, we tested whether the formulations were able to induce cellular senescence in the CCA cell line. Although C1 did not decrease the number of CCA cells as shown with CVSA, this compound triggered a cellular senescence response that was comparable to the one induced by gemcitabine (Supplementary Figure S17A). Combination with gemcitabine showed a synergistic effect and more senescence was detected in all tested combination groups (Supplementary Figure S17A). We performed an additional quantitative FACS-based assay to detect C_12_FDG-stained senescent cells (gating strategy is shown in Supplementary Figure S17B). The obtained results strongly correlated with SA-β-Gal assay results and confirmed that C1 (concentrations 4 and 8 mg/ml) combined with gemcitabine increased senescence (Supplementary Figure S17A,C). Interestingly, in contrast to C1, C2 induced a weaker senescence response (Supplementary Figure S17D) whereas C3 induced a stronger senescence response which was comparable or more pronounced than in the gemcitabine group (Supplementary Figure S17E).

#### 3.2.3 C2 induced late apoptosis and necroptosis while inhibiting the cholangiocarcinoma cell line as detected *via* flow cytometry analysis

**FIGURE 9:**
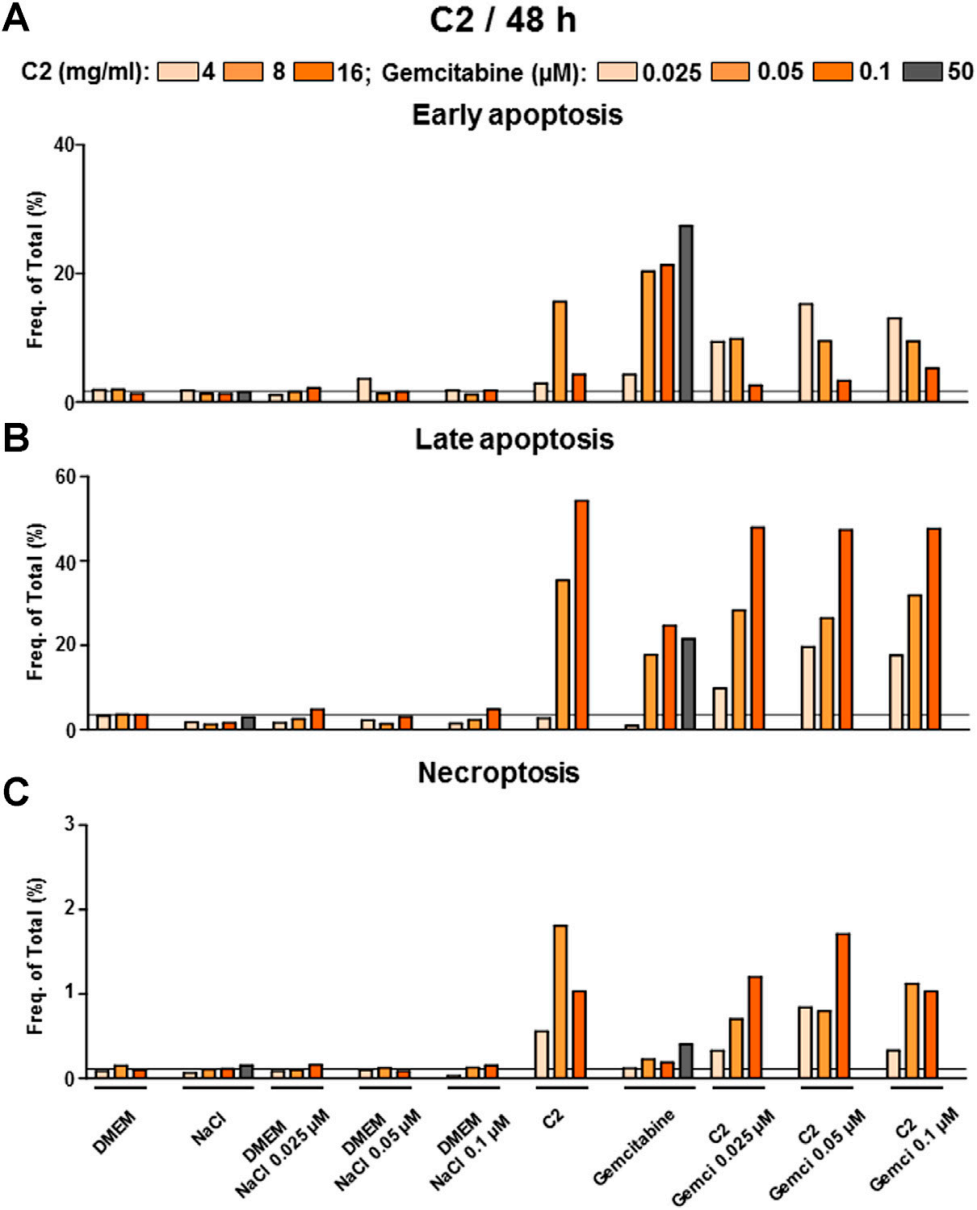
C2 alone and in combination with gemcitabine induced early, late apoptosis and necroptosis in CCA cells. FACS analysis to detect **(A)** early, **(B)** late apoptosis and **(C)** necroptosis was performed in C2-treated CCA cells. Shown are frequencies in percent. The grey line represents the values of the control group (DMEM).

**FIGURE 10:**
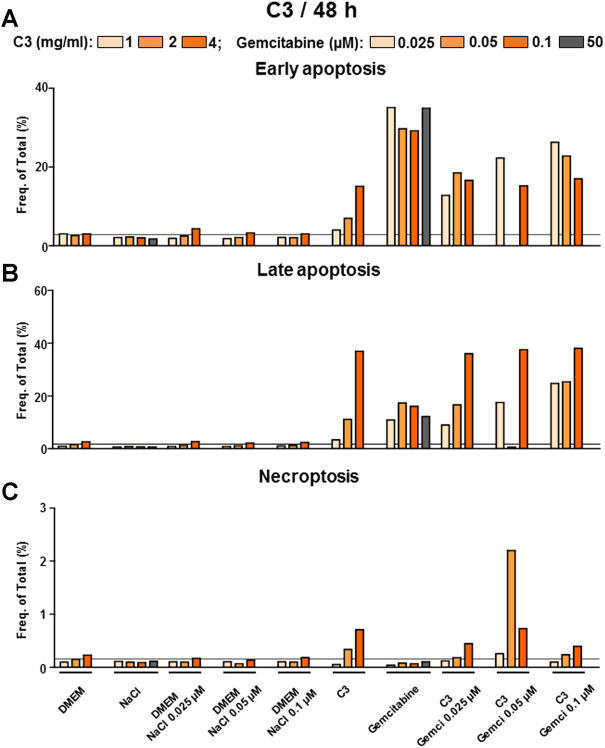
C3 alone and in combination with gemcitabine induced mostly early and late apoptosis in CCA cells. FACS analysis to detect **(A)** early, **(B)** late apoptosis and **(C)** necroptosis was performed in C3-treated CCA cells. Shown are frequencies in percent. The grey line represents the values of the control group (DMEM).

We further searched for the mechanism of inhibition investigating early and late apoptosis as well as necroptosis upon treatment with compounds (Supplementary Figure S18 for C1, Figure 9 for C2 and Figure 10 for C3). Standard chemotherapeutic gemcitabine induced early and late apoptosis and very little necroptosis (Supplementary Figure S18; Figures 9, 10). In contrast to the HCC data, C1 in CCA induced mostly 1) late apoptosis that was much lower than the one shown with the gemcitabine human plasma concentration (50 µM) and 2) necroptosis superior to gemcitabine group (Supplementary Figure S18). In contrast to C1, both highly active compounds in CCA, C2 and C3, induced early and late apoptosis. In early apoptosis, C2 and C3 were less efficient than gemcitabine human plasma concentration (Figures 9A, 10A). However, in late apoptosis, monotherapeutic and combination regimes of C2 and C3 showed a striking efficacy in comparison to gemcitabine human plasma concentration (Figures 9B, 10B). Similar to HCC data, C2 also demonstrated a clear induction of necroptosis, whereas C3 did not show such efficacy in all tested formulations (Figures 9C, 10C). Interestingly, when combinations were applied in C2 or C3 groups, they did not show any stronger early or late apoptosis compared to the gemcitabine monotherapy (Figure 10), which does not correlate with CVSA data.

## 4 Discussion

In this study, we investigated the inhibitory properties of TCM (C1) and TTM (C2 and C3) in two types of PLC, HCC and CCA, *in vitro.* Standard chemotherapeutics which are currently used in the clinic (sorafenib for HCC and gemcitabine for CCA) were used as positive controls. A detailed LC-MS/MS analysis of C1–C3 provided an overview of the mainly hydrophilic to mid-polar character of all compounds with C2 having the most mid-polar complexity. The chromatographic trace of C1 was similar compared to a previous study (Hu et al., 2016). Importantly, to our knowledge, LC-MS/MS characterizations of extracts from C2 and C3 have not been described in literature so far, and are presented for the first time in this study.

In our research, we investigated the compounds applied as monotherapies or in combination with reduced doses of chemotherapeutics. Importantly, both chemotherapeutics induce strong side effects in patients. Sorafenib, a standard medicine for HCC treatment since 2007, is accompanied by adverse side effects including fatigue, diarrhea, and skin dermatologic toxicity. Combination therapy aimed to lower side effects seems to be an approach with high perspectives (Hochnadel et al., 2017; Raoul et al., 2019; Walcher et al., 2020). Moreover, combination therapies using sorafenib and TCM were often hypothesized as promising (Cao et al., 2020; Zhou et al., 2020).

A study comparing the safety and efficacy of Huaier and sorafenib monotherapies following HCC resection demonstrated no significant differences between both tested drugs. However, the study showed fewer side effects in patients treated with Huaier (Jianyong et al., 2014). Importantly, in the sorafenib-treated group even a discontinuation of sorafenib was required due to severe adverse reactions in patients in comparison to Huaier-treated patients (Jianyong et al., 2014). Further, a multicenter randomized clinical trial has proven the effectiveness of Huaier granules as an adjuvant therapy after curative liver resection and resulted in significant prolongation of recurrence-free survival and reduced extrahepatic recurrence rates. The average concentration of sorafenib in plasma after standard administration reaches 13.8 μM (Fucile et al., 2015). The results of our investigation have shown that in combination C1 or C3 with even a half dose of sorafenib (6.9 μM) was sufficient to inhibit the growth of HCC cells. Such combination approaches can be especially important for patients who do not tolerate the full dose of sorafenib and take only a half dose due to strong side effects of the drug (Dufour et al., 2010). C3 applied at 1 mg/ml also showed efficacy alone without sorafenib. C2 did not inhibit the HCC cell line alone but in combination with sorafenib (3.45 and 6.9 μM). This TTM formulation increased the therapeutic effect of standard anti-cancer medicine.

Previous studies reported, that traditional Chinese herbal medicine on example of a multi-component Long-Dan-Xie-Gan-Tang formulation does not affect sorafenib metabolism in animal models (Ting et al., 2017). This allows for the hypothesis that combination therapy will benefit both from the curative effect of sorafenib and traditional preparations. Thus, in clinical practice, transarterial chemoembolization (TACE) combined with C1 treatment has already been used in HCC therapy (Zhao et al., 2017). Huaier extract has been reported to improve the health status of HCC patients after resection or liver transplantation (Lei et al., 2015; Chen et al., 2018). Our research hypothesis is further supported by other studies, showing promising results of combinations of Huaier in HCC and other cancers. Such, combination treatment of oxaliplatin and Huaier had a significant synergistic anti-cancer effect and inhibited expression of Yes-associated protein (Tao et al., 2018). In another study, Huaier granules combined with Tegafur Gimeracil Oteracil Potassium could promote patient prognosis with a better disease-free and overall survival rate and induced apoptosis in gastric cancer (Qi et al., 2020a). Additionally, Huaier aqueous extract combined with routine chemotherapeutic drugs showed a synergistic effect on human acute lymphoblastic leukemia cells *in vitro* (Qu et al., 2020).

Huaier is known to decrease the proliferative and migratory potential of HCCs in murine models partially by down-regulation of Yes-associated protein 1 (Shan et al., 2017; Tao et al., 2018), to decrease the levels of phosphorylated AKT and mTOR (Bao et al., 2016), and to interfere in liver tumor angiogenesis (Li et al., 2015; Qi et al., 2020b). Still, the molecular mechanisms of HCC inhibition need further investigation.

In our study, we detected that two compounds C1 and C3 were the most efficacious in HCC, whereas in CCA C2 and C3 compounds demonstrated strong inhibitory properties and C1 unexpectedly proved to be inefficient. In contrast to our observation on C1 in CCA, a case study report of a CCA patient receiving hepatectomy and subsequent treatment with C1 showed an improvement in recurrence-free survival, indicating that C1 has the potential to be used for the treatment of CCA (Feng et al., 2022). Although C2 showed no effect on HCC in our study, Ngamkitidechakul et al. (2010), Achari et al. (2011), and Zhang et al. (2021) reported the effect of the single components of C2, showing its potency to inhibit HCC. Interestingly, C2 and C3 share four common ingredients (*T. chebula* Retz., *A. debilis* Siebold & Zuccarini, *Meconopsis cambrica* Vig., *B. testilis* McClure). We assume that these four components were responsible for the efficacy of C2 and C3 in CCA, where both compounds showed strong efficacy. In line with our research, several studies investigated these four components as a single agent in different types of cancer and confirmed their efficacy in the inhibition of cancer cell growth (Saleem et al., 2002; Achari et al., 2011; Fan et al., 2015a; Fan et al., 2015b; Ravi Shankara et al., 2016). C3 showed to be effective in both types of cancer in our study. It´s efficacy in HCC is probably mediated by the combination of three ingredients that are exclusive for C3 (are not present in C2 which was inefficient in HCC) and comprise: *Carthamus tinctorius* L., *Swertia bimaculata,* and *Ephedra sinica* Stapf. Notably, the efficacy of those three single components of C3 was reported in other types of cancer (Park et al., 2016; Sharula and Wu, 2017; Hyuga et al., 2020) and is further supported by our study.

Importantly, combinations of the successful compounds with standard chemotherapeutics demonstrated the most pronounced efficacy in comparison to human plasma concentrations of standard chemotherapeutics. We observed that the results of CVSA and CCK-8 assays detecting the inhibition of cell growth strongly correlated. We could show that late apoptosis was the main mechanism beyond the inhibition of C1 and C3 in HCC and early and late apoptosis represented the main protective mechanism of C2 and C3 in CCA. It is worth to mention, that our data on the mechanism of sorafenib standard chemotherapy in HCC and gemcitabine standard chemotherapy in CCA is fully in line with previous reports from Lin and colleagues (Lin et al., 2017) and Pauwels et al. (2009) and Li et al. (2020b), respectively, as well as the recent report by Pylypchuk et al. (2022).

In line with our studies, treatment of human HCC cell lines HepG2 and Huh7 with Huaier extracts resulted in apoptosis development (Bao et al., 2016; Xu et al., 2020).

We further demonstrated that cellular senescence and necroptosis were not associated with the inhibition of HCC and CCA cells in our study. Cycle arrest of the cells and subsequently entering the cellular senescence program is beneficial for limiting liver cancer development, as we have previously shown (Kang et al., 2011; Yevsa et al., 2012; Eggert et al., 2016; Hochnadel et al., 2017; Walcher et al., 2020). Senescent cells are characterized by a special senescence-associated secretory phenotype (so called “SASP”) including production and secretion of pro-inflammatory cytokines and chemokines influencing cell-cell interactions and tissue homeostasis (Lee and Schmitt, 2019) and attracting immune cells towards senescence sites (Kang et al., 2011; Eggert et al., 2016). Therapy-induced senescence plays an important role in suppression of cancer development (Yevsa et al., 2012; Hochnadel et al., 2017; Walcher et al., 2020; Wyld et al., 2020). There are several reports demonstrating that Huaier is able to induce senescence in cancerous cells. Huaier extract increased amount of cells in S and G2/M phases in gastric cancer cell lines (Wang et al., 2019), induced G0/G1 arrest in endocrine resistant breast cancer cell lines (Gao et al., 2017) and HCC cell lines (Bao et al., 2016), and S phase arrest in hepatoblastoma cells (Xu et al., 2020). Our study is in line with previous reports, as we observed, that C1 as a monotherapy and a combination therapy with sorafenib induced cellular senescence in HCC and CCA cell lines. However, we did not observe, that senescence correlated with protective capacity and inhibition of HCC and CCA *in vitro*. We do not exclude that induction of senescence *in vivo* might result in a very strong immune response towards senescent cancerous cells, which will further increase protection against liver cancer, as recently reviewed (Walcher et al., 2020).

It is important to mention that molecular mechanisms of multicomponent C2 and C3 in HCC and CCA are highly unanswered and there is no information in scientific literature about the application of C2 and C3 for CCA treatment. A few studies showing their effects on cholestatic hepatitis (Yang, 2000; Ci et al., 2018), alcoholic liver injury and chronic liver injury induced by CCl_4_ (Ma et al., 2020) have been published. Although several single constituents of C2 and C3 have been reported to inhibit the growth of cancerous cells in different types of cancer (as reviewed by us in this study in Supplementary Tables S5–S7), the reports for multicomponent C2 and C3 are still very scarce. Our research has deepened the knowledge on C2 and C3. We defined the mechanism and found out that C3 as a monotherapy or in combination with 3.45 or 6.9 μM of sorafenib decreased the HCC cell line growth, thereby inducing cellular senescence and late apoptosis. However, C2 was efficient only in combination with sorafenib and induced late apoptosis and necroptosis in HCC.

Our study provides further knowledge on mechanisms beyond the efficacy of C1 in CCA. It was reported that a combination of Huaier with 5-fluorouracil (5-FU) *in vitro* on the CCA cell line Huh28 resulted in growth inhibition, apoptosis and arrest of cells in the S phase (Fu et al., 2019). Inhibition of proliferation, metastasis and invasion of CCA cells by activating apoptosis and downregulating TP73-AS1 in CCA after the treatment with Huaier have been described (Ji et al., 2020). In contrast to other reports, C1 extract in our experiments did not reduce the amount of CCA cells. The low efficacy of C1 in CCA is probably associated with the induction of cellular senescence as well as necroptosis phases, whereas protective early and late apoptosis were less efficient than in the gemcitabine group.

Side effects of gemcitabine include fatigue, nausea and vomiting, diarrhea, leuco- and thrombopenia and allergic reactions. Severe cases including cardio-pulmonary insufficiency with fatal consequences have been also described (Tonato et al., 1995; Shan et al., 2017). Taking into account the poor prognosis for CCA patients (Banales et al., 2020), various possibilities of combination therapy have to be urgently investigated. Successful combination therapy with Huaier and gemcitabine was described for pancreatic cancer but not for CCA (Chen et al., 2021). In contrast, we found that C1 was not efficient in CCA inhibition. However, we observed a strong inhibitory effect of C2 and C3 on CCA cell line growth and the efficacy was dependent on the induction of early and late apoptosis.

Summarizing everything mentioned above, we could state for the first time that the mechanism of inhibitory effect of C2 and C3 on CCA is explained by triggering early and late apoptosis, whereas cellular senescence and necroptosis did not correlate with protection.

Combination therapy of C2 or C3 with gemcitabine on CCA allowed decreasing the effective concentration of this standard medicine from 50 μM (human plasma concentration) to 0.1, 0.05 or even 0.025 μM.

One of the most important outcomes from combination therapy is a possibility to reduce the therapeutic doses of toxic anti-cancer preparations. Addition of TCM and TTM to schemes of the standard therapy could allow decreasing the consumption of anticancer drugs with the same efficacy of treatment. To confirm this suggestion, further experiments *in vivo* using C1, C2 and C3 in autochthonous HCC and CCA murine models need to be performed.

## 5 Conclusion

In this research, we studied the inhibitory properties of TCM (C1) and TTM (C2 and C3) in two types of PLC, HCC and CCA, *in vitro*. We detected, that two compounds C1 and C3 were the most efficacious in HCC, whereas in CCA C2 and C3 compounds demonstrated strong inhibitory properties and C1 was unexpectedly shown as inefficient. Importantly, combinations of the successful compounds with standard chemotherapeutics (sorafenib for HCC and gemcitabine for CCA) demonstrated the most pronounced efficacy in comparison to human plasma concentrations of standard chemotherapeutics. Moreover, application of combination therapy allowed for a strong reduction of standard chemotherapeutics: a half dose of sorafenib or even a dose 500 fold lower of gemcitabine applied were more efficacious in combination with compounds than human plasma concentrations of standard chemotherapeutics. The mechanism behind the successful inhibition was associated with the induction of late apoptosis in HCC and early/late apoptosis in CCA (see graphical abstract). Cellular senescence and necroptosis do not seem to be involved in the inhibition of HCC and CCA cell lines *in vitro*. Further *in vivo* studies using autochthonous HCC and CCA models are required to evaluate the efficacy of TCM/TTM and mechanism behind it. Special importance has to be given to combination therapies which show synergistic effects, allowing for a reduction of toxic chemotherapeutics and have therefore a strong perspective for patients with HCC and CCA.

## Data Availability

The original contributions presented in the study are included in the article/Supplementary Material, further inquiries can be directed to the corresponding author.
