# Supplementary material for: Elucidating the mechanism behind and investigating the efficacy of Traditional Chinese Medicine and Traditional Tibetan Medicine in combination with standard therapeutics in hepatocellular carcinoma and cholangiocarcinoma in vitro
Source: Front Pharmacol. 2022 Sep 12;13:906468. doi: 10.3389/fphar.2022.906468 (PMC9511410; doi:10.3389/fphar.2022.906468)
Supplement: Supplementary file 2 [file DataSheet1.pdf]

Supplementary Material

List of Abbreviations

**Annexin V-PE**	Annexin V-phycoerythrin
**7-AAD**	7-amino-actinomycin D
**C1**	Compound 1 (in this study *Trametes robiniophila* Murr, Huaier)
**C2**	Compound 2 (in this study Ershiwuwei Songshi Wan)
**C3**	Compound 3 (in this study Qiwei Honghua Shusheng Wan)
**C_{12}FDG**	5-Dodecanoylaminofluorescein di-β-D-galactopyranoside
**CCA**	Cholangiocarcinoma
**CCK-8**	Cell counting kit – 8
**CVSA**	Crystal violet staining assay
**DMEM**	Dulbecco's Modified Eagle Medium
**DMSO**	Dimethyl sulfoxide
**FACS**	Fluorescence-activated cell sorting (flow cytometry analysis)
**FBS**	Fetal bovine serum
**5-FU**	5-Fluorouracil
**HCC**	Hepatocellular carcinoma
**iCCA**	Intrahepatic cholangiocarcinoma
**LC-MS/MS**	Liquid chromatography mass spectrometry
**MEM NEAA**	Minimum essential medium non-essential amino acids
**MQ**	Milli-Q
**NaCl**	Sodium chloride
**N.A.**	Not applicable
**OD**	Optical density
**PBS**	Phosphate buffered saline
**PLC**	Primary liver cancer
**SA-β-Gal**	Senescence-associated β-galactosidase assay

**SASP**	Senescence-associated secretory phenotype
**SEM**	Standard error of the mean
**PLC**	Primary liver cancer
**TACE**	Transarterial chemoembolization
**TCM**	Traditional Chinese Medicine
**TTM**	Traditional Tibetan Medicine

Supplementary Tables

Supplementary Table 1 | List of all species contained in the formulations of C1, C2 and C3.

<table>
<tr><td colspan="3">C1: Huaier
Link to the purchaser: www.gaitianli.com.cn</td></tr>
<tr><th colspan="2">Ingredient</th><th>Content in formulation</th></tr>
<tr><td colspan="2">Trametes robiniophila Murr</td><td>100%</td></tr>
<tr><td colspan="3">C2: Ershiwuwei Songshi Wan
Link to the purchaser: www.glzy.cn</td></tr>
<tr><th>No.</th><th>Ingredient</th><th>Content in formulation (g)</th></tr>
<tr><td>1</td><td>Turquoise (mineral)</td><td>20</td></tr>
<tr><td>2</td><td>Pearl</td><td>10</td></tr>
<tr><td>3</td><td>Corals</td><td>40</td></tr>
<tr><td>4</td><td>Asparagaceae: Dracaena cinnabari Balf. f.</td><td>20</td></tr>
<tr><td>5</td><td>Combretaceae: Terminalia chebula Retz.</td><td>50</td></tr>
<tr><td>6</td><td>Combretaceae: Scrap iron of Terminalia chebula Retz.</td><td>100</td></tr>
<tr><td>7</td><td>Phyllanthaceae: Phyllanthus emblica L.</td><td>50</td></tr>
<tr><td>8</td><td>Sciuridae: Trogopterus xanthipes (Minlne Eduards)</td><td>40</td></tr>
<tr><td>9</td><td>Santalaceae: Santalum album L.</td><td>40</td></tr>
<tr><td>10</td><td>Fabaceae: Dalbergia odorifera T. C. Chen</td><td>40</td></tr>
<tr><td>11</td><td>Aristolochiaceae: Aristolochia debilis Siebold & Zucc.</td><td>50</td></tr>
</table>

12	Acanthaceae: *Justicia adhatoda* L.	50
13	Bovidae: *Bos taurus domesticus* Gmelin	5
14	Asteraceae: *Dolomiaea costus* (Falc.) Kasana & A. K. Pandey	60
15	Papaveraceae: *Meconopsis cambrica* Vig.	50
16	Ranunculaceae: *Aconitum naviculare* (Brühl) Stapf	40
17	Myristicaceae: *Myristica fragrans* Gronov	20
18	Myrtaceae: *Syzygium aromaticum* (L.) Merr. & L. M. Perry	25
19	Saxifragaceae*: Saxifraga umbellulata* var. *pectinata* (C. Marquand & Airy Shaw) J. T. Pan	50
20	Combretaceae: *Terminalia bellirica* (Gaertn.) Roxb.	5
21	Poaceae*: Bambusa testilis* McClure	35
22	Iridaceae: *Crocus sativus* L.	5
23	Malvaceae: *Bombax ceiba* L.	35
24	Moschidae: *Moschus* Flerov	0.25
25	Travertine (limestone)	35
**C3: Qiwei Honghua Shusheng Wan**<br>Link to the purchaser: www.glzy.cn		
**No.**	**Ingredient**	**Content in formulation (g)**
1	Asteraceae: *Carthamus tinctorius* L.	112.5
2	*Poaceae: Bambusa testilis* McClure	75
3	Gentianaceae: *Swertia bimaculata* (Siebold & Zucc.) Hook. f. & Thomson ex C. B. Clarke	75
4	Combretaceae: *Terminalia chebula* Retz.	100
5	Ephedraceae: *Ephedra sinica* Stapf	75
6	Aristolochiaceae: *Aristolochia debilis* Siebold & Zucc.	75
7	Papaveraceae: *Meconopsis cambrica* Vig.	75

Supplementary Table 2 | The concentrations of TCM, TTM and standard therapeutics used in this study.

Cancer Type	Compounds				
	**C1 (mg/ml)**	**C2 (mg/ml)**	**C3 (mg/ml)**	**Sorafenib (µM)**	**Gemcitabine (µM)**
**HCC**	4<br>8<br>16	1<br>2<br>4	0.25<br>0.5<br>1	3.45<br>6.9<br>13.8<br>(human plasma concentration)	Not applicable (N.A.)
**CCA**	4<br>8<br>16	4<br>8<br>16	1<br>2<br>4	N.A.	0.025<br>0.05<br>0.1<br>50<br>(human plasma concentration)

Supplementary Table 3 | Individual metabolites detected in C1, C2 and C3. Table 3 is attached as a separate Excel file.

Supplementary Table 4 | Statistical analysis of all treatment groups of TCM, TTM and standard therapeutic agents in HCC and CCA representing *P* values. Table 4 is attached as a separate Excel file.

Supplementary Table 5 | Cancer studies describing inhibitory capacity of C1.

Constituent	Type of cancer	Investigated in human, *in vivo*, *in vitro*	Reference
Huaier *(Trametes robiniophila* Murr*)*	Breast cancer	MDA-MB-231, MCF7<br>Mouse study	(Wang et al., 2019)
	HCC	Patients<br><br>Rabbit study and HepG2 cells	(Chen et al., 2018; J.Y.Lei, 2015; Wang et al., 2021)<br><br>(Ren et al., 2009)<br><br>(Shan et al., 2017)

		Bel-7404, Bel-7402, SMMC-7721	
	Gastric cancer	Mouse study and MGC803, MKN74, AZ-521, MKN28 cells	(Shi et al., 2022)
	CCA	Patients<br><br>Huh28<br><br>RBE, CCLP1, HuCCT1	(Feng et al., 2022)<br><br>(Fu et al., 2019)<br><br>(Ji et al., 2020)

Supplementary Table 6 | Cancer studies describing inhibitory capacity of individual constituents of C2.

**Constituent**	**Type of cancer**	**Investigated cells/animals**	**Reference**
Turquoise (mineral)	N.A.	N.A.	N.A.
Pearl	N.A.	N.A.	N.A.
Corals	Breast cancer	SKBR3, MDA-MB-231	(Huang et al., 2018)
	HCC	SK-HEP-1	(Ko et al., 2021)
Dracaena cinnabari Balf. f.	N.A.	N.A.	N.A.
[§]*Terminalia chebula* Retz.	Breast cancer	MCF-7, S115 (mouse)	(Saleem et al., 2002)
	Osteosarcoma	HOS-1	
	Prostate cancer	PC-3	
	Mammary gland adenocarcinoma<br>Lung cancer	MCF-7<br>A-549	(Ravi Shankara et al., 2016)
	HCC	HepG2	(Achari et al., 2011)
Scrap iron of *Terminalia chebula* Retz.	N.A.	N.A.	N.A.
Phyllanthus emblica L.	Lung cancer<br>HCC<br><br>Cervical cancer<br>Breast cancer<br>Ovarian cancer<br>Colorectal cancer	A-549<br>HepG2<br><br>HeLa<br>MDA-MB-231<br>SK-OV3<br>SW620	(Ngamkitidechakul et al., 2010)

	Lung cancer<br>HCC	A-549<br>HepG2	(Pinmai et al., 2008)
	Colorectal cancer	Colo205	(Guo et al., 2017)
Trogopterus xanthipes (Minlne Eduards)	N.A.	N.A.	N.A.
Santalum album L.	Bladder cancer	J82	(Dozmorov et al., 2014)
	Skin cancer	A431	(Kaur et al., 2005)
	Prostate cancer	PC-3	(Bommareddy et al., 2012)
Dalbergia odorifera T.C. Chen	Cervical cancer	C-33A, SiHa	(Yang et al., 2018)
§*Aristolochia debilis* Siebold & Zuccarini	N.A.	N.A.	N.A.
Justicia adhatoda L.	T-cell lymphoma	Mouse study	(V., 2019)
	Cervical cancer	HeLa	(Pandiyan et al., 2019; Sudevan et al., 2019)
Bos taurus domesticus Gmelin	HCC	SMMC-7721	(Dimin et al., 2022; Zhang et al., 2021)
Dolomiaea costus (Falc.) Kasana & A. K. Pandey	N.A.	N.A.	N.A.
§*Meconopsis cambrica* Vig.	Lymphocytic leukemia	L1210	(Fan et al., 2015b)
	Leukemia	K562	(Fan et al., 2015a)
Aconitum naviculare (Brühl) Stapf	N.A.	N.A.	N.A.
Myristica fragrans Gronov	Sarcoma	Mouse study	(Thuong et al., 2014)
	Oral epidermal carcinoma	Oral epidermal carcinoma KB cells	(Rengasamy, 2018)
Syzygium aromaticum (L.) Merr. & L. M. Perry	Colon carcinoma	Human colon carcinoma cells	(Yassin, 2020)
Saxifraga umbellulata var. pectinata (C. Marquand & Airy Shaw) J. T. Pan	N.A.	N.A.	N.A.
Terminalia bellirica (Gaertn.) Roxb.	Breast ductal carcinoma<br>Colorectal cancer<br>Colon cancer<br>Breast cancer<br>Ductal carcinoma<br>Prostatic cancer	BT-474<br>Colo-205<br>HAT-29<br>MCF-7<br>ZR-75-1<br>LnCap	(Shi Li, 2018)
§*Bambusa testilis* McClure	Breast cancer	Rat study	(Lin et al., 2008)
Crocus sativus L.	HCC	Rat study	(Amin et al., 2011)
	HCC<br>Cervical cancer	HepG2<br>HeLa	(Tavakkol-Afshari et al., 2008)

Bombax ceiba L.	Mammary gland carcinoma	Rat study	(Mahmoud, 2020)
	Leukemia	HL-60	(Sharma et al., 2020)
Moschus Flerov	Lung cancer<br><br><br><br><br>Mammary carcinoma<br><br>Esophageal carcinoma<br><br>Gastric carcinoma<br><br>Colorectal carcinoma<br><br><br>HCC<br>Acute myelogenous leukemia<br>B cell lymphoma	Eplc-32M1, GLC-82, XLA-07, XL-JT, A549, NCIH-460, 801-D, NCIH-446<br>MDA-MB-231<br><br>TE-1<br><br>HSC, NCI-N87SGC-7901<br>HT-29, Caco-2SW480<br><br>Huh7, HepG2,<br>HL-60<br><br>Daudi	(Xu and Cao, 2016)
Travertine (limestone)	N.A.	N.A.	N.A.

§These compounds are also represented in C3.

Supplementary Table 7 | Cancer studies describing inhibitory capacity of individual constituents of C3.

Constituent	Type of cancer	Investigated cells/animals	Reference
Carthamus tinctorius L.	Colorectal cancer<br><br>Breast cancer	HCT116, SW480<br>LoVo, HT-29<br><br>MDA-MB-231<br>MCF-7	(Park, 2016)
	HCC	HepG2, Hep3B	(Sharula and Wu, 2017)
§§*Bambusa testilis* McClure	Breast cancer	Rat study	(Lin et al., 2008)
Swertia bimaculata (Siebold & Zucc.) Hook. f. & Thomson ex C.B. Clarke	N.A.	N.A.	N.A.
§§*Terminalia chebula* Retz.	Breast cancer<br>Osteosarcoma<br>Prostate cancer	MCF-7, S115,<br>HOS-1<br>PC-3	(Saleem et al., 2002)

	Mammary gland adenocarcinoma<br>Lung cancer	MCF-7<br><br>A-549	(Ravi Shankara et al., 2016)
	HCC	HepG2	(Achari et al., 2011)
Ephedra sinica Stapf	Lung cancer	H1993	(Hyuga et al., 2020)
§§*Aristolochia debilis* Siebold & Zucc.	N.A.	N.A.	N.A.
§§*Meconopsis cambrica* Vig.	Lymphocytic leukemia	L1210	(Fan et al., 2015b)
	Leukemia	K562	(Fan et al., 2015a)

§§These compounds are also represented in C2.

Supplementary Figure Legends

Supplementary Figure 1 | A detailed description of the 25 components of the TTM C2 used in the study. 25 components of C2 comprise of the following: 1) Turquoise; 2) Pearl; 3) Corals; 4) *Dracaena cinnabari* Balf. f.; 5) *Terminalia chebula* Retz.; 6) Scrap iron of *Terminalia chebula* Retz.; 7) *Phyllanthus emblica* L.; 8) *Trogopterus xanthipes* (Minlne Eduards); 9) *Santalum album* L.; 10) *Dalbergia odorifera* T.C. Chen; 11) *Aristolochia debilis* Siebold & Zuccarini; 12) *Justicia adhatoda* L.; 13) *Bos taurus domesticus* Gmelin; 14) *Dolomiaea costus* (Falc.) Kasana & A. K. Pandey; 15) *Meconopsis cambrica* Vig.; 16) *Aconitum naviculare* (Brühl) Stapf; 17) *Myristica* fragrans Gronov; 18) *Syzygium aromaticum* (L.) Merr. & L.M.Perry; 19) *Saxifraga umbellulata var. pectinata* (C. Marquand & Airy Shaw) J.T. Pan; 20) *Terminalia bellirica* (Gaertn.) Roxb.; 21) *Bambusae concertio silicea*; 22) *Crocus sativus* L.; 23) *Bombax ceiba* L.; 24) *Moschus* Flerov; 25) Travertine (limestone). The flower symbol represents a plant-origin.

Supplementary Figure 2 | A detailed description of the seven components of the TTM C3 used in this study. The seven components comprise: 1) *Carthamus tinctorius* L.; 2) *Bambusa testilis* McClure; 3) *Swertia bimaculata* (Siebold & Zucc.) Hook. F. & Thomson ex C. B. Clarke; 4) *Terminalia chebula* Retz.; 5) *Ephedra sinica* Stapf; 6) *Aristolochia debilis* Siebold & Zucc; 7) *Meconopsis cambrica* Vig. The flower symbol represents a plant-origin.

Supplementary Figure 3 | Analysis of C1, C2 and C3 components by **LC-MS/MS**. The powders C1, C2 and C3 were extracted in water and analyzed by ultrahigh-performance liquid chromatography coupled to a quadrupol time-of-flight mass spectrometry in positive mode. The figure shows the base peak chromatograms for C1 to C3 and a blank sample (top to bottom).

Supplementary Figure 4 | C1 possessed inhibitory capacity towards HCC cell line as confirmed by CVSA analysis using ImageJ. Cell viability analysis performed **(A)** 24 h and **(B)** 48 h post-incubation. Values represent mean ± standard error of the mean (Petriv et al.). Significance levels were depicted as: *$p < 0.05$, **$p < 0.01$, ***$p < 0.001$ and ****$p < 0.0001$. Detailed *P* values for all groups are displayed in **Supplementary Table 4**.

Supplementary Figure 5 | C2 in combination with sorafenib possessed an inhibitory capacity towards the HCC cell line in CVSA analysis, whereas C2 monotherapy was completely inefficient. CVSA readouts were performed **(A)** 24 h and **(B)** 48 h after incubation to test the inhibitory capacity of C2 at

concentrations 1, 2, 4 mg/ml as a monotherapy or in combination with sorafenib (3.45 µM and 6.9 µM). Sorafenib dose of 13.8 µM (plasma concentration, as reported for the clinic (Fucile et al., 2015)), has been used as a positive control. DMSO and DMEM were used as negative controls, as a carrier for sorafenib and C2, respectively.

Supplementary Figure 6 | C2 possessed inhibitory capacity towards HCC cell line as confirmed by CVSA analysis using ImageJ. Cell viability analysis performed **(A)** 24 h and **(B)** 48 h post-incubation. Values represent mean ± SEM. Significance levels were depicted as: *$p < 0.05$, **$p < 0.01$, ***$p < 0.001$ and ****$p < 0.0001$. Detailed *P* values for all groups are displayed in **Supplementary Table 4**.

Supplementary Figure 7 | C3 in combination with sorafenib inhibited the growth of the HCC cell line, as shown by CVSA analysis. CVSA has been performed **(A)** 24 h and **(B)** 48 h after incubation, to test the inhibitory capacity of C3 at concentrations 0.25, 0.5 and 1 mg/ml as monotherapy or in combination with sorafenib (3.45 µM and 6.9 µM). DMSO and DMEM were used as negative controls, as a carrier for sorafenib and C3, respectively.

Supplementary Figure 8 | C3 possessed inhibitory capacity towards HCC cell line as confirmed by CVSA analysis using ImageJ. Cell viability analysis performed **(A)** 24 h and **(B)** 48 h post-incubation. Values represent mean ± SEM. Significance levels were depicted as: *$p < 0.05$, **$p < 0.01$, ***$p < 0.001$ and ****$p < 0.0001$. Detailed *P* values for all groups are displayed in **Supplementary Table 4**.

Supplementary Figure 9 | C3 demonstrated an inhibitory effect on the HCC cell line both as a monotherapy and in combination with sorafenib in CCK-8 analysis and induced cellular senescence. CCK-8 analysis was performed on **(Martinez-Velez et al.)** C2- or **(C-D)** C3-treated HCC cells. OD_{450} was detected **(A-C)** 24 h and **(B-D)** 48 h after incubation with compounds. Values represent mean ± SEM. Significance levels were depicted as: *$p < 0.05$, **$p < 0.01$ and ****$p < 0.0001$. **(E)** SA-β-Gal assay was performed 48 h post-incubation in C3-treated group. Senescent cells stained in blue are depicted with the white arrows.

Supplementary Figure 10 | FACS analysis performed 48 h after incubation revealed the induction of late apoptosis in HCC cells treated with C2 combined with sorafenib. FACS analysis to detect **(A)** early, **(B)** late apoptosis and **(C)** necroptosis was performed and results are shown as frequencies in percent. The grey line represents the values for the control group (DMEM).

Supplementary Figure 11 | FACS analysis performed 48 h after the incubation with C3 alone or in combination with sorafenib confirmed the induction of late apoptosis in HCC cells. FACS analysis to detect **(A)** early, **(B)** late apoptosis and **(C)** necroptosis was performed and results are shown as frequencies in percent. The grey line represents the values for the DMEM control group.

Supplementary Figure 12 | C1 showed no impact on CCA cell line growth in CVSA after 24 h of incubation. CVSA has been performed to test the inhibitory capacity of C1 alone or in combination with sorafenib 24 h post-incubation. Carriers (DMEM for C1, NaCl for gemcitabine) were used as negative controls. Gemcitabine was applied at different concentrations increasing from 0.025, 0.05, 0.1 to 50 µM (human plasma concentration (Fujiwara et al., 2015)), which was applied as a positive control.

Supplementary Figure 13 | C1 shows no impact on CCA cell line growth in CVSA after 48 h of incubation. CVSA has been performed to test the inhibitory capacity of C1 alone or in combination with sorafenib 48 h post-incubation as described in **Supplementary Figure 12**.

Supplementary Figure 14 | C1 possessed inhibitory capacity towards CCA cell line as confirmed by CVSA analysis using ImageJ. Cell viability analysis performed **(A)** 24 h and **(B)** 48 h post-incubation. Values represent mean ± SEM. Significance levels were depicted as: $*p < 0.05$, $**p < 0.01$, $***p < 0.001$ and $****p < 0.0001$. Detailed *P* values for all groups are displayed in **Supplementary Table 4**.

Supplementary Figure 15 | C2 possessed inhibitory capacity towards CCA cell line as confirmed by CVSA analysis using ImageJ. Cell viability analysis performed **(A)** 24 h and **(B)** 48 h post-incubation. Values represent mean ± SEM. Significance levels were depicted as: $*p < 0.05$, $**p < 0.01$, $***p < 0.001$ and $****p < 0.0001$. Detailed *P* values for all groups are displayed in **Supplementary Table 4**.

Supplementary Figure 16 | C3 possessed inhibitory capacity towards CCA cell line as confirmed by CVSA analysis using ImageJ. Cell viability analysis performed **(A)** 24 h and **(B)** 48 h post-incubation. Values represent mean ± SEM. Significance levels were depicted as: $*p < 0.05$, $**p < 0.01$, $***p < 0.001$ and $****p < 0.0001$. Detailed *P* values for all groups are displayed in **Supplementary Table 4**.

Supplementary Figure 17 | C1 and C3 induced a stronger cellular senescence response than gemcitabine as detected by SA-β-Gal assay and FACS analysis. **(A)** SA-β-gal assay was performed to detect senescent cells in C1-treated CCA cells 48 h post-incubation. **(B)** Gating strategy to detect $C_{12}FDG^+$ senescent, early and late apoptotic, and necroptotic cells. Cells were gated using forward- and side scatter characteristics and duplicates were omitted. Thereafter $C_{12}FDG^+$ (FDG^+) senescent cells were detected and three different populations based on 7-AAD and Annexin V-PE staining were detected among FDG^- cells. **(C-D)** Detection of FDG^+ senescent CCA cells treated with **(C)** C1, **(D)** C2 and **(E)** C3. The grey line represents the values for the control group DMEM.

Supplementary Figure 18 | C1 induced mostly necroptosis and late apoptosis in CCA cells as shown by FACS analysis. FACS analysis to detect **(A)** early, **(B)** late apoptosis and **(C)** necroptosis was performed and shown as frequencies in percent. The grey line represents the values for the DMEM control group.

Supplementary References

Achari, C., Reddy, G.V., Reddy, T.C., and Reddanna, P. (2011). Chebulagic acid synergizes the cytotoxicity of doxorubicin in human hepatocellular carcinoma through COX-2 dependant modulation of MDR-1. Med Chem *7*, 432-442.

Amin, A., Hamza, A.A., Bajbouj, K., Ashraf, S.S., and Daoud, S. (2011). Saffron: a potential candidate for a novel anticancer drug against hepatocellular carcinoma. Hepatology *54*, 857-867.

Bommareddy, A., Rule, B., VanWert, A.L., Santha, S., and Dwivedi, C. (2012). alpha-Santalol, a derivative of sandalwood oil, induces apoptosis in human prostate cancer cells by causing caspase-3 activation. Phytomedicine *19*, 804-811.

Chen, Q., Shu, C., Laurence, A.D., Chen, Y., Peng, B.G., Zhen, Z.J., Cai, J.Q., Ding, Y.T., Li, L.Q., Zhang, Y.B., *et al.* (2018). Effect of Huaier granule on recurrence after curative resection of HCC: a multicentre, randomised clinical trial. Gut *67*, 2006-2016.

Dozmorov, M.G., Yang, Q., Wu, W., Wren, J., Suhail, M.M., Woolley, C.L., Young, D.G., Fung, K.M., and Lin, H.K. (2014). Differential effects of selective frankincense (Ru Xiang) essential oil versus non-selective sandalwood (Tan Xiang) essential oil on cultured bladder cancer cells: a microarray and bioinformatics study. Chin Med *9*, 18.

Fan, J., Wang, P., Wang, X., Tang, W., Liu, C., Wang, Y., Yuan, W., Kong, L., and Liu, Q. (2015a). Induction of Mitochondrial Dependent Apoptosis in Human Leukemia K562 Cells by Meconopsis integrifolia: A Species from Traditional Tibetan Medicine. Molecules *20*, 11981-11993.

Fan, J., Wang, Y., Wang, X., Wang, P., Tang, W., Yuan, W., Kong, L., and Liu, Q. (2015b). The antitumor activity of Meconopsis horridula Hook, a traditional Tibetan medical plant, in murine leukemia L1210 cells. Cell Physiol Biochem *37*, 1055-1065.

Feng, J.Y., Li, X.P., Wu, Z.Y., Ying, L.P., Xin, C., Dai, Z.Z., Shen, Y., and Wu, Y.F. (2022). Sarcomatoid intrahepatic cholangiocarcinoma with good patient prognosis after treatment with Huaier granules following hepatectomy: A case report. World J Clin Cases *10*, 2829-2835.

Fu, Z., Ma, K., Dong, B., Zhao, C., Che, C., Dong, C., Zhang, R., Wang, H., Wang, X., and Liang, R. (2019). The synergistic antitumor effect of Huaier combined with 5-Florouracil in human cholangiocarcinoma cells. BMC Complement Altern Med *19*, 203.

Guo, X.H., Ni, J., Xue, J.L., and Wang, X. (2017). Phyllanthus emblica Linn. fruit extract potentiates the anticancer efficacy of mitomycin C and cisplatin and reduces their genotoxicity to normal cells in vitro. J Zhejiang Univ Sci B *18*, 1031-1045.

Huang, H.W., Tang, J.Y., Ou-Yang, F., Wang, H.R., Guan, P.Y., Huang, C.Y., Chen, C.Y., Hou, M.F., Sheu, J.H., and Chang, H.W. (2018). Sinularin Selectively Kills Breast Cancer Cells Showing G2/M Arrest, Apoptosis, and Oxidative DNA Damage. Molecules *23*.

Hyuga, S., Hyuga, M., Amakura, Y., Yang, J., Mori, E., Hakamatsuka, T., Goda, Y., Odaguchi, H., and Hanawa, T. (2020). Effect of Ephedra Herb on Erlotinib Resistance in c-Met-Overexpressing Non-Small-Cell Lung Cancer Cell Line, H1993, through Promotion of Endocytosis and Degradation of c-Met. Evidence-Based Complementary and Alternative Medicine *2020*, 7184129.

J.Y.Lei, L.N.Y., J.Q. Zhu, W.T.Wang (2015). Hepatocellular Carcinoma Patients May Benefit From Postoperative Huaier Aqueous Extract After Liver Transplantation. Transplantation Proceedings *47*, 2920-2924.

Ji, D., Zheng, W., Huang, P., Yao, Y., Zhong, X., Kang, P., Wang, Z., Shi, G., Xu, Y., and Cui, Y. (2020). Huaier Restrains Cholangiocarcinoma Progression in vitro and in vivo Through Modulating lncRNA TP73-AS1 and Inducing Oxidative Stress. Onco Targets Ther *13*, 7819-7837.

Kaur, M., Agarwal, C., Singh, R.P., Guan, X., Dwivedi, C., and Agarwal, R. (2005). Skin cancer chemopreventive agent, {alpha}-santalol, induces apoptotic death of human epidermoid carcinoma A431 cells via caspase activation together with dissipation of mitochondrial membrane potential and cytochrome c release. Carcinogenesis *26*, 369-380.

Ko, C.Y., Shih, P.C., Huang, P.W., Lee, Y.H., Chen, Y.F., Tai, M.H., Liu, C.H., Wen, Z.H., and Kuo, H.M. (2021). Sinularin, an Anti-Cancer Agent Causing Mitochondria-Modulated Apoptosis and Cytoskeleton Disruption in Human Hepatocellular Carcinoma. Int J Mol Sci *22*.

Lin, Y., Collier, A.C., Liu, W., Berry, M.J., and Panee, J. (2008). The inhibitory effect of bamboo extract on the development of 7,12-dimethylbenz[a]anthracene (DMBA)-induced breast cancer. Phytother Res *22*, 1440-1445.

Mahmoud, A.H., Metwally, N.S., Youness, E.R., El- Toukhy, S.E., Elmalt, H.A., Al-Mokaddem, A.K. (2020). Antiproliferative Activity of Bombax ceiba Flower Extract against Mammary Gland Carcinoma in Rats. Systemic Reviews in Pharmacy *11*, 1406-1415.

Martinez-Velez, N., Garcia-Moure, M., Marigil, M., Gonzalez-Huarriz, M., Puigdelloses, M., Gallego Perez-Larraya, J., Zalacain, M., Marrodan, L., Varela-Guruceaga, M., Laspidea, V.*, et al.* (2019). The oncolytic virus Delta-24-RGD elicits an antitumor effect in pediatric glioma and DIPG mouse models. Nat Commun *10*, 2235.

Ngamkitidechakul, C., Jaijoy, K., Hansakul, P., Soonthornchareonnon, N., and Sireeratawong, S. (2010). Antitumour effects of Phyllanthus emblica L.: induction of cancer cell apoptosis and inhibition of in vivo tumour promotion and in vitro invasion of human cancer cells. Phytother Res *24*, 1405-1413.

Ning Dimin, D.Z., Wu Yongrong, Mei Si, Teng Yongjie, Zhou Qing, Tian Xuefei (2022). Niuhuang (Bovis Calculus)-Shexiang (Moschus) combination induces apoptosis and inhibits proliferation in hepatocellular carcinoma via PI3K/AKT/mTOR pathway. Digital Chinese Medicine *5*, 83-92.

Pandiyan, N., Murugesan, B., Arumugam, M., Sonamuthu, J., Samayanan, S., and Mahalingam, S. (2019). Ionic liquid - A greener templating agent with Justicia adhatoda plant extract assisted green synthesis of morphologically improved Ag-Au/ZnO nanostructure and it's antibacterial and anticancer activities. J Photochem Photobiol B *198*, 111559.

Park, G.H., Hong, S.C., Jeong, J. B. (2016). Anticancer Activity of the Safflower Seeds (Carthamus tinctorius L.) through Inducing Cyclin D1 Proteasomal Degradation in Human Colorectal Cancer Cells. Korean Journal of Plant Resources *29*, 297-304.

Petriv, N., Neubert, L., Vatashchuk, M., Timrott, K., Suo, H., Hochnadel, I., Huber, R., Petzold, C., Hrushchenko, A., Yatsenko, A.S.*, et al.* (2021). Increase of α-dicarbonyls in liver and receptor for advanced glycation end products on immune cells are linked to nonalcoholic fatty liver disease and liver cancer. OncoImmunology *10*, 1874159.

Pinmai, K., Chunlaratthanabhorn, S., Ngamkitidechakul, C., Soonthornchareon, N., and Hahnvajanawong, C. (2008). Synergistic growth inhibitory effects of Phyllanthus emblica and Terminalia bellerica extracts with conventional cytotoxic agents: doxorubicin and cisplatin against human hepatocellular carcinoma and lung cancer cells. World J Gastroenterol *14*, 1491-1497.

Ravi Shankara, B.E., Ramachandra, Y.L., Rajan, S.S., Ganapathy, P.S., Yarla, N.S., Richard, S.A., and Dhananjaya, B.L. (2016). Evaluating the Anticancer Potential of Ethanolic Gall Extract of Terminalia chebula (Gaertn.) Retz. (Combretaceae). Pharmacognosy Res *8*, 209-212.

Ren, J., Zheng, C., Feng, G., Liang, H., Xia, X., Fang, J., Duan, X., and Zhao, H. (2009). Inhibitory effect of extract of fungi of Huaier on hepatocellular carcinoma cells. J Huazhong Univ Sci Technolog Med Sci *29*, 198-201.

Rengasamy, G., Venkataraman, A., Veeraraghavan, V.P, Jainu M. (2018). Cytotoxic and apoptotic potential of Myristica fragrans Houtt. (mace) extract on human oral epidermal carcinoma KB cell lines. Brazilian Journal of Pharmaceutical Sciences.

Saleem, A., Husheem, M., Harkonen, P., and Pihlaja, K. (2002). Inhibition of cancer cell growth by crude extract and the phenolics of Terminalia chebula retz. fruit. J Ethnopharmacol *81*, 327-336.

Shan, L., Li, Y., Jiang, H., Tao, Y., Qian, Z., Li, L., Cai, F., Ma, L., and Yu, Y. (2017). Huaier Restrains Proliferative and Migratory Potential of Hepatocellular Carcinoma Cells Partially Through Decreased Yes-Associated Protein 1. J Cancer *8*, 4087-4097.

Sharma, N., Kispotta, S., and Mazumder, P. (2020). Immunomodulatory and anticancer activity of <i>Bombax ceiba</i> Linn leaf extract. Asian Pacific Journal of Tropical Biomedicine *10*, 426-432.

Sharula, and Wu, Z. (2017). Regulation of Apoptosis by SYB in HepG2 Liver Cancer Cells is Mediated by the P53/Caspase 9 Axis. Anticancer Agents Med Chem *17*, 941-947.

Shi Li, T.Y., Linjin Liang, Wenyi Liang, Ping Jian, Kun Zhou, Lanzhen Zhang (2018). Anti-cancer activity of an ethyl-acetate extract of the fruits of *Terminalia bellerica* (Gaertn.) Roxb. through an apoptotic signaling pathway *in vitro*. Journal of Traditional Chinese Medical Sciences *5*, 370-379.

Shi, Y., Yuan, L., Xu, J., Xu, H., Wang, L., Huang, L., Xu, Z., and Cheng, X. (2022). Huaier Inhibits Gastric Cancer Growth and Hepatic Metastasis by Reducing Syntenin Expression and STAT3 Phosphorylation. J Oncol *2022*, 6065516.

Sudevan, S., Parasivam, R., Sundar, S., Velauthan, H., and Ramasamy, V. (2019). Investigation of anti-inflammatory and anti-cancer activity of Justicia adathoda metabolites. Pak J Pharm Sci *32*, 1555-1561.

Tavakkol-Afshari, J., Brook, A., and Mousavi, S.H. (2008). Study of cytotoxic and apoptogenic properties of saffron extract in human cancer cell lines. Food Chem Toxicol *46*, 3443-3447.

Thuong, P.T., Hung, T.M., Khoi, N.M., Nhung, H.T., Chinh, N.T., Quy, N.T., Jang, T.S., and Na, M. (2014). Cytotoxic and anti-tumor activities of lignans from the seeds of Vietnamese nutmeg Myristica fragrans. Arch Pharm Res *37*, 399-403.

V., J. (2019). Assessment of Invivo Anticancer Activity of Justicia Adathoda Using Dal Cell Lines. East African Scholars Journal of Medical Sciences *2*, 438-442.

Wang, W., Wang, X., Li, C., Chen, T., Zhang, N., Liang, Y., Li, Y., Zhang, H., Liu, Y., Song, X., *et al.* (2019). Huaier Suppresses Breast Cancer Progression via linc00339/miR-4656/CSNK2B Signaling Pathway. Front Oncol *9*, 1195.

Wang, Z., Yu, X.L., Zhang, J., Cheng, Z.G., Han, Z.Y., Liu, F.Y., Dou, J.P., Kong, Y., Dong, X.J., Zhao, Q.X., *et al.* (2021). Huaier granule prevents the recurrence of early-stage hepatocellular carcinoma after thermal ablation: A cohort study. J Ethnopharmacol *281*, 114539.

Xu, L., and Cao, Y. (2016). Native musk and synthetic musk ketone strongly induced the growth repression and the apoptosis of cancer cells. BMC Complement Altern Med *16*, 511.

Yang, P.Y., Hu, D.N., Kao, Y.H., Lin, I.C., and Liu, F.S. (2018). Butein induces apoptotic cell death of human cervical cancer cells. Oncol Lett *16*, 6615-6623.

Yassin, M.T., Al-Askar, A.A., Abdel-Fattah, A., El-Sheikh, M.M.A. (2020). Bioactivity of Syzygium aromaticum (L.) Merr. & L.M.Perry extracts as potential antimicrobial and anticancer agents. Journal of King Saud University - Science *32*, 3273-3278.

Zhang, Z., Zeng, P., Gao, W., Wu, R., Deng, T., Chen, S., and Tian, X. (2021). Exploration of the Potential Mechanism of Calculus Bovis in Treatment of Primary Liver Cancer by Network Pharmacology. Comb Chem High Throughput Screen *24*, 129-138.
