# Supplementary material for: Elucidating the mechanism behind and investigating the efficacy of Traditional Chinese Medicine and Traditional Tibetan Medicine in combination with standard therapeutics in hepatocellular carcinoma and cholangiocarcinoma in vitro
Source: Front Pharmacol. 2022 Sep 12;13:906468. doi: 10.3389/fphar.2022.906468 (PMC9511410; doi:10.3389/fphar.2022.906468)
Supplement: Supplementary file 4 [file Image1.pdf]

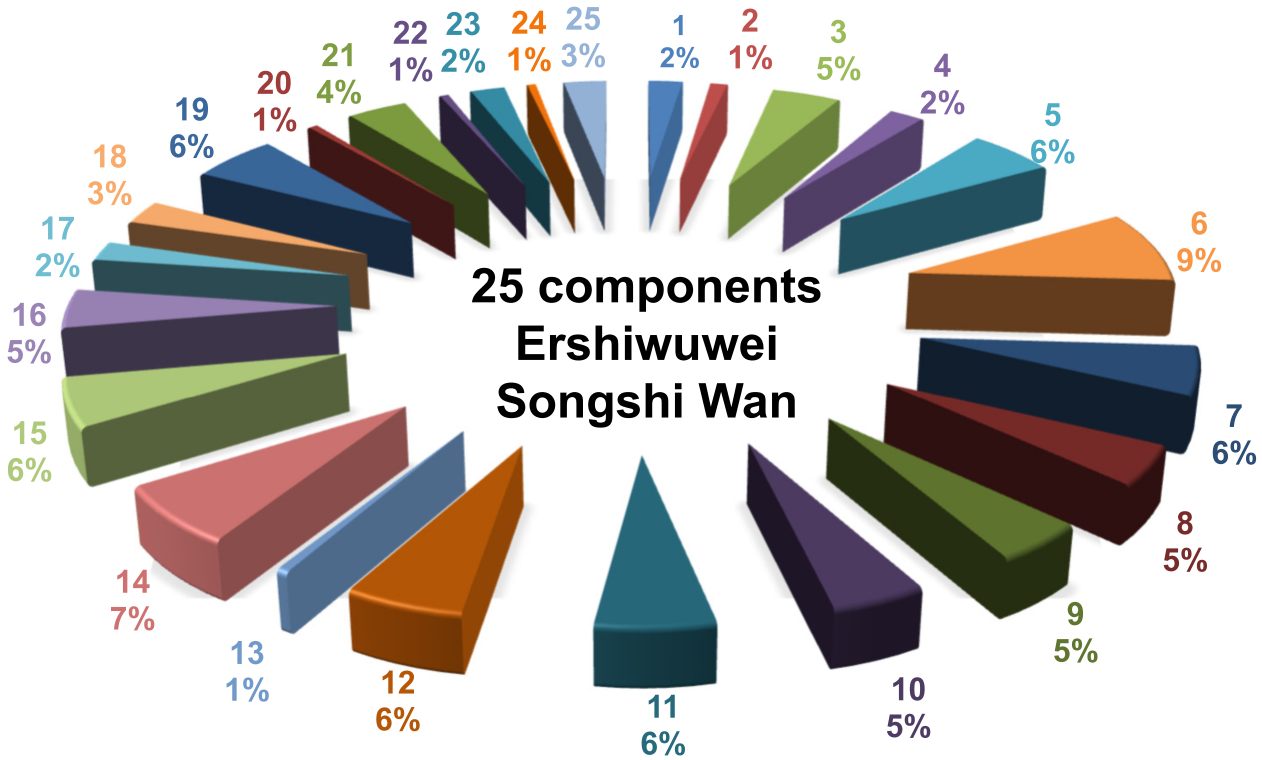

No	Ingredient	
1	Turquoise (mineral)	
2	Pearl	
3	Corals	
4	Asparagaceae: *Dracaena cinnabari* Balf. f.	
5	Combretaceae: *Terminalia chebula* Retz.	❋
6	Combretaceae: Scrap iron of *Terminalia chebula* Retz.	❋
7	Phyllanthaceae: *Phyllanthus emblica* L.	❋
8	Sciuridae: *Trogopterus xanthipes* (Minlne Eduards)	
9	Santalaceae: *Santalum album* L.	❋
10	Fabaceae: *Dalbergia odorifera* T. C. Chen	❋
11	Aristolochiaceae: *Aristolochia debilis* Siebold & Zucc.	❋
12	Acanthaceae: *Justicia adhatoda* L.	❋
13	Bovidae: *Bos taurus domesticus* Gmelin	
14	Asteraceae: *Dolomiaea costus* (Falc.) Kasana & A. K. Pandey	❋
15	Papaveraceae: *Meconopsis cambrica* Vig.	❋

No	Ingredient	
16	Ranunculaceae: *Aconitum naviculare* (Brühl) Stapf	❋
17	Myristicaceae: *Myristica fragrans* Gronov	❋
18	Myrtaceae: *Syzygium aromaticum* (L.) Merr. & L. M. Perry	❋
19	Saxifragaceae*: Saxifraga umbellulata* var. *pectinata* (C. Marquand & Airy Shaw) J. T. Pan	❋
20	Combretaceae: *Terminalia bellirica* (Gaertn.) Roxb.	❋
21	Poaceae*: Bambusa testilis* McClure	
22	Iridaceae: *Crocus sativus* L.	❋
23	Malvaceae: *Bombax ceiba* L.	❋
24	Moschidae: *Moschus* Flerov	
25	Travertine (limestone)	

Plant-origin ❋

Supplementary Figure 1

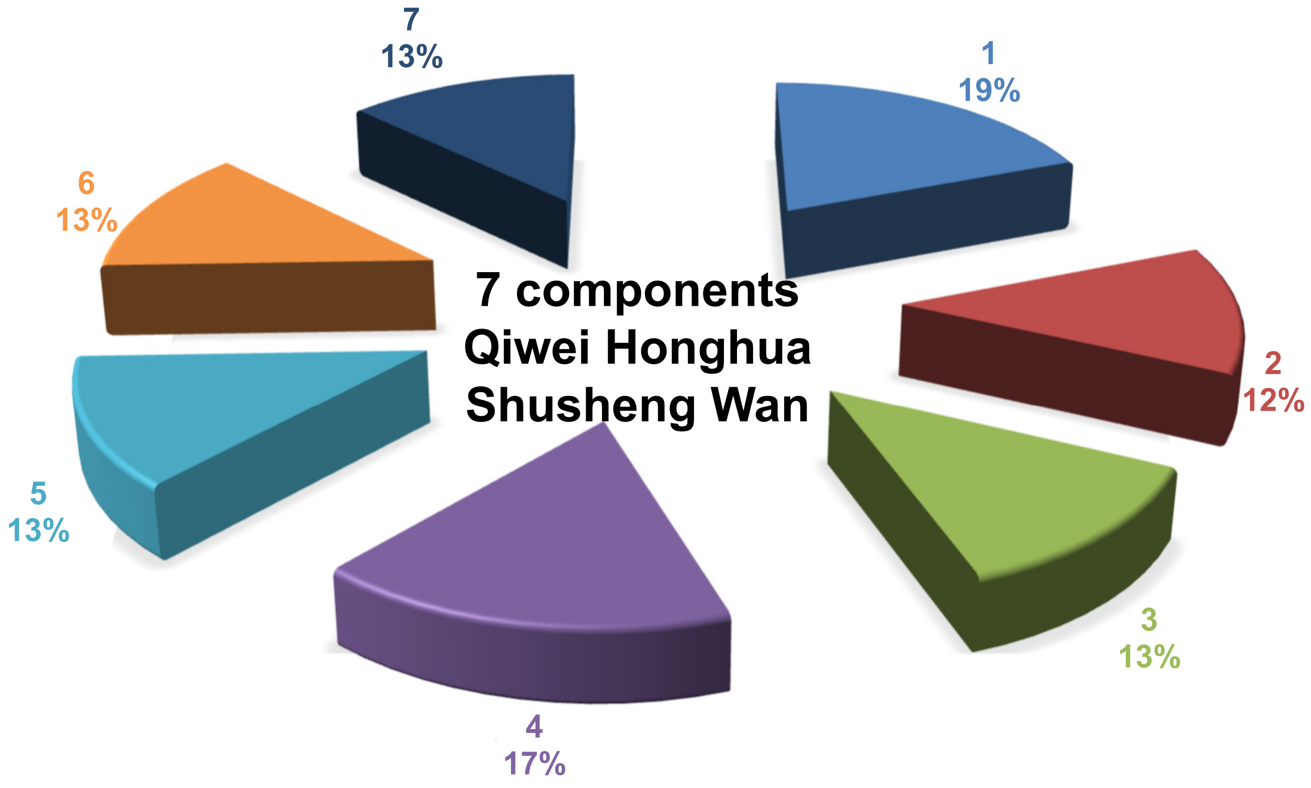

No	Ingredient
1	Asteraceae: *Carthamus tinctorius* L. ❋
2	*Poaceae: Bambusa testilis* McClure
3	Gentianaceae: *Swertia bimaculata* (Siebold & Zucc.) Hook. f. & Thomson ex C. B. Clarke ❋
4	Combretaceae: *Terminalia chebula* Retz.
5	Ephedraceae: *Ephedra sinica* Stapf ❋
6	Aristolochiaceae: *Aristolochia debilis* Siebold & Zucc. ❋
7	Papaveraceae: *Meconopsis cambrica* Vig. ❋

Plant-origin ❋

Supplementary Figure 2

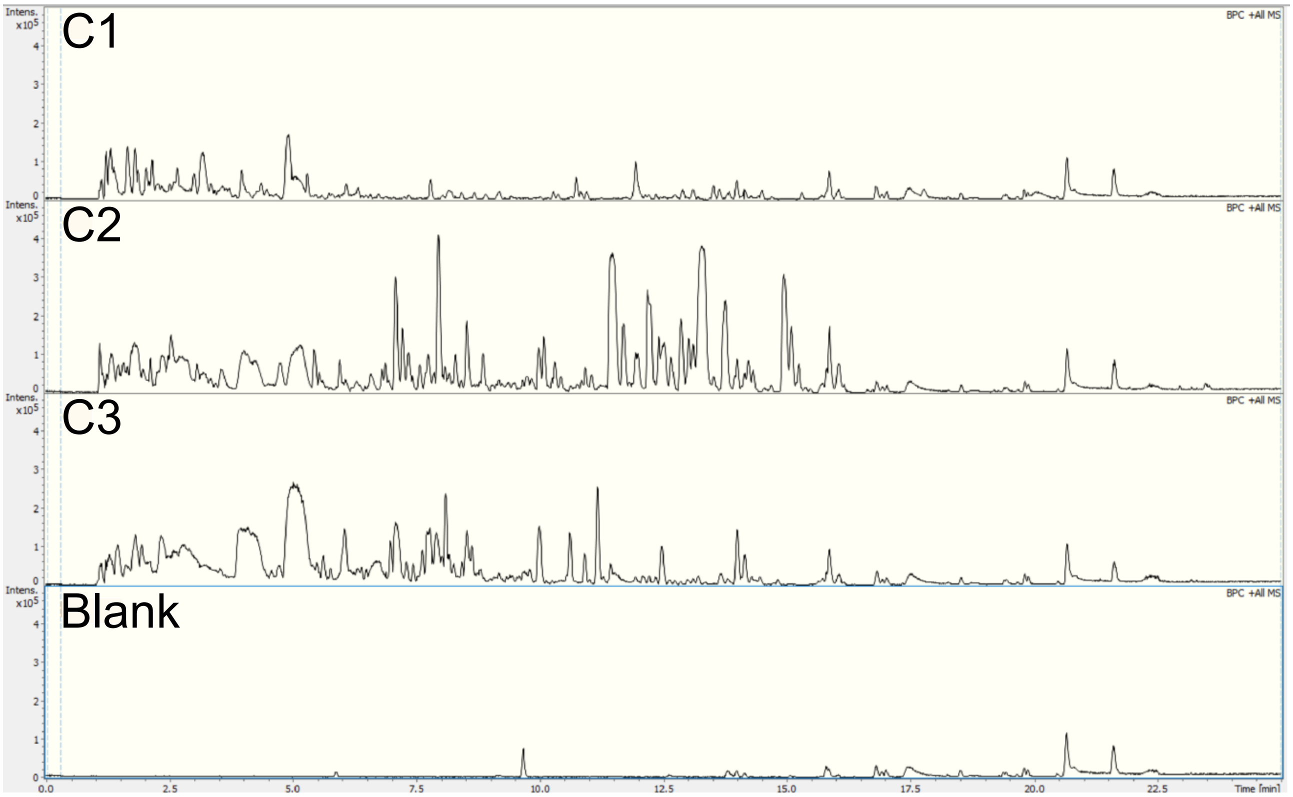

Supplementary Figure 3

(A)

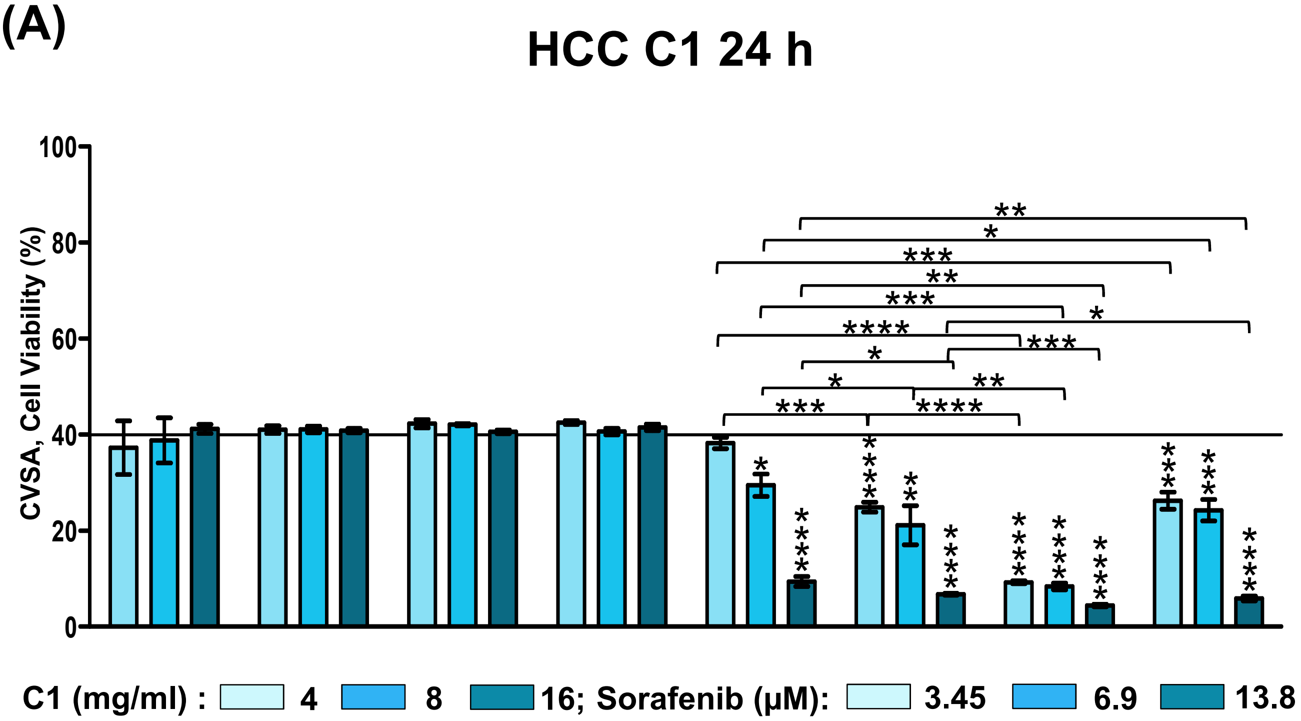

(B)

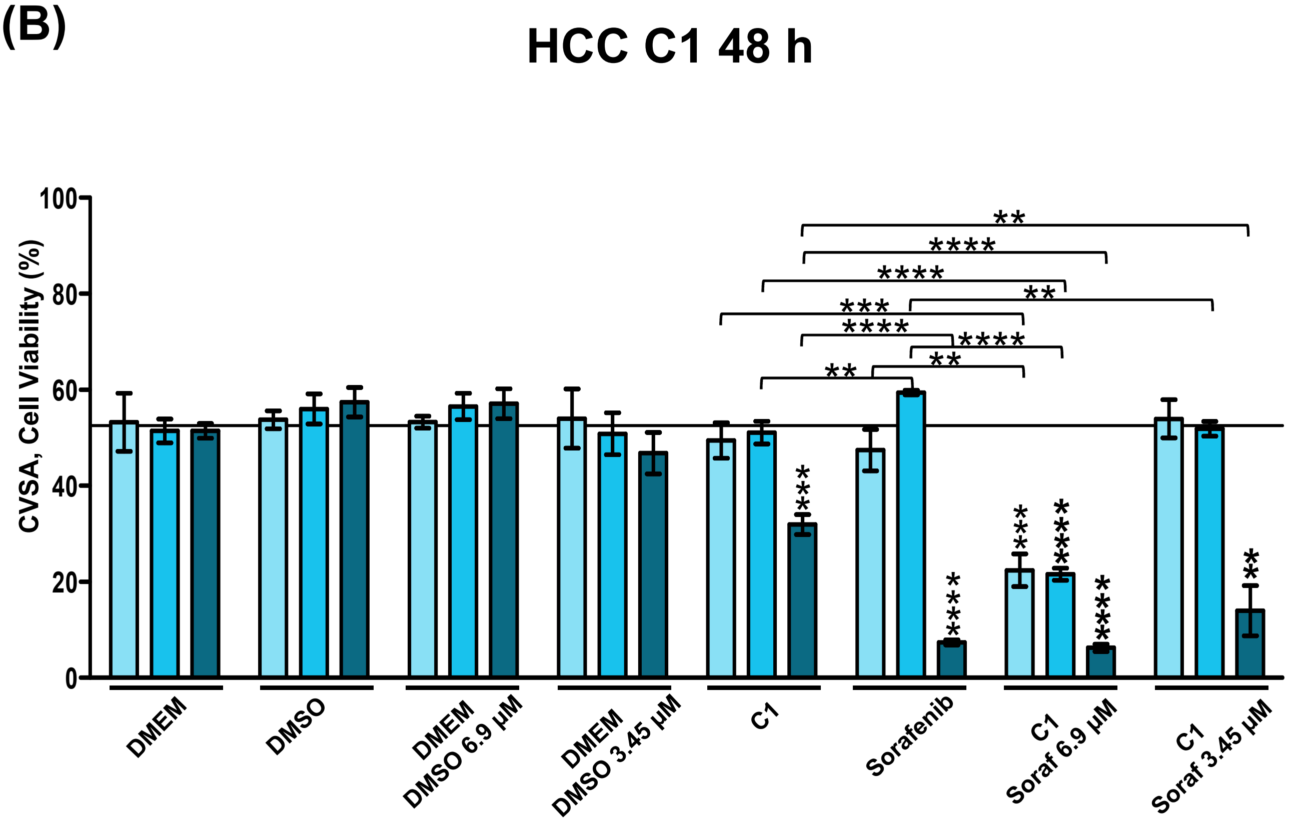

Supplementary Figure 4

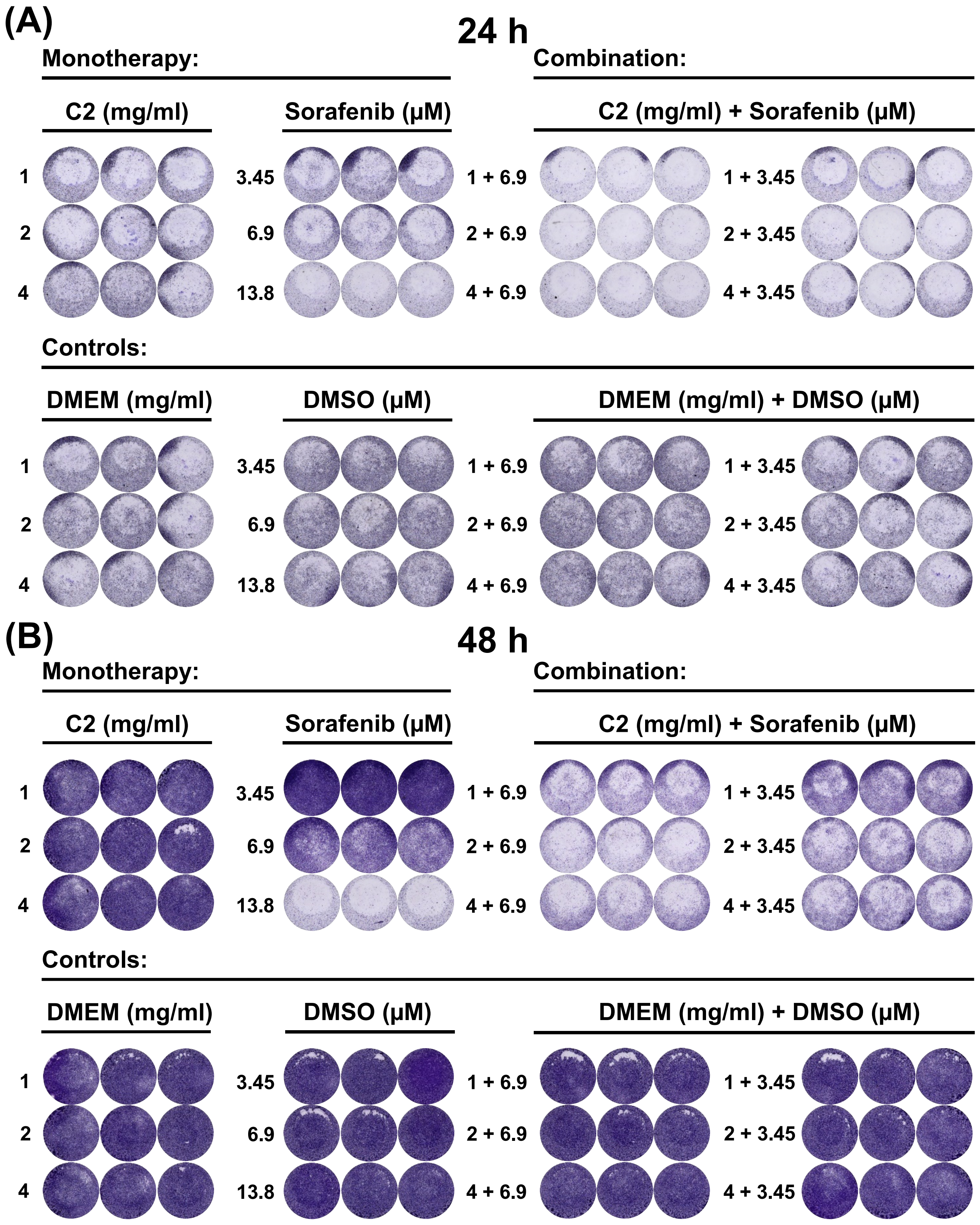

Supplementary Figure 5

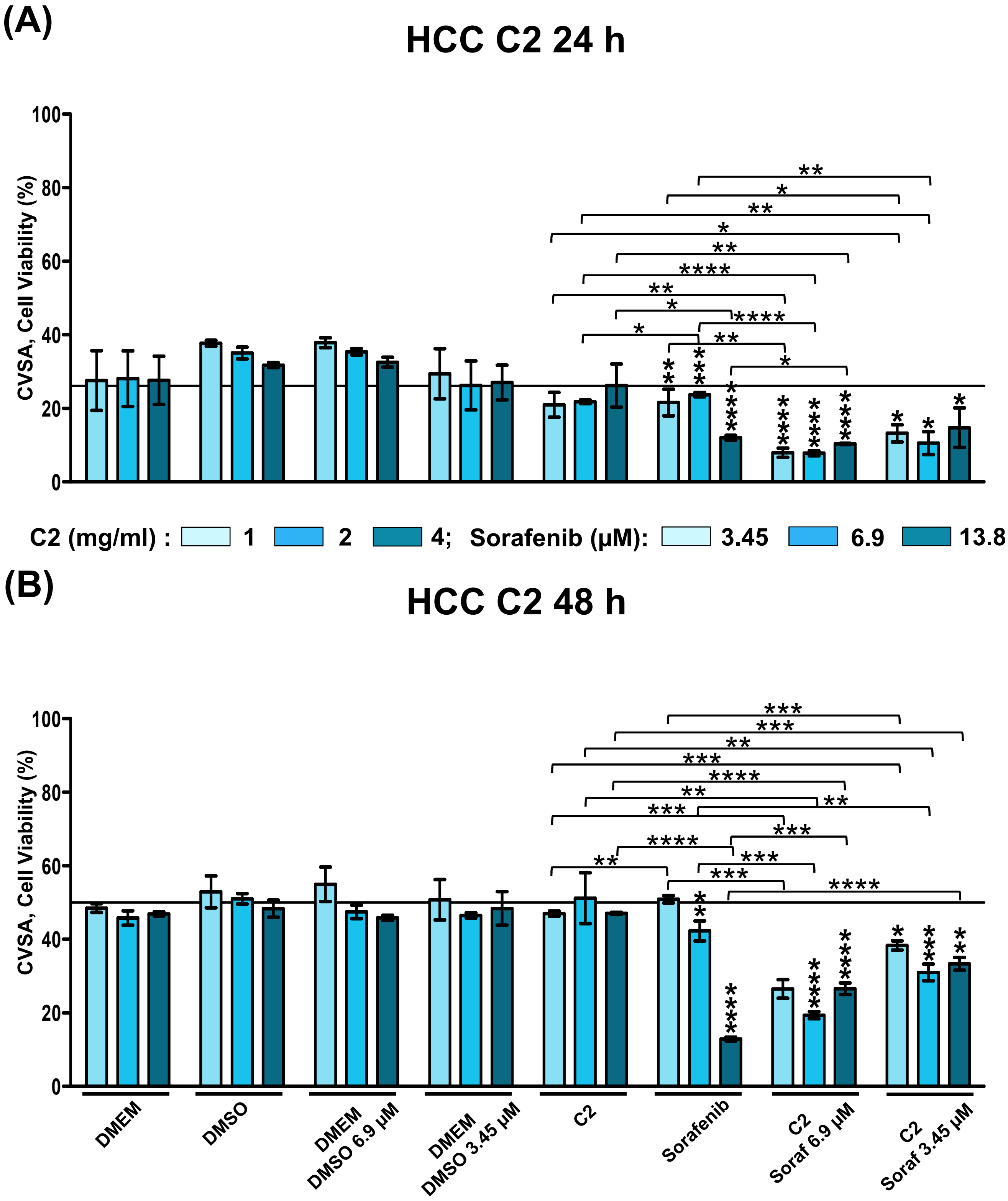

Supplementary Figure 6

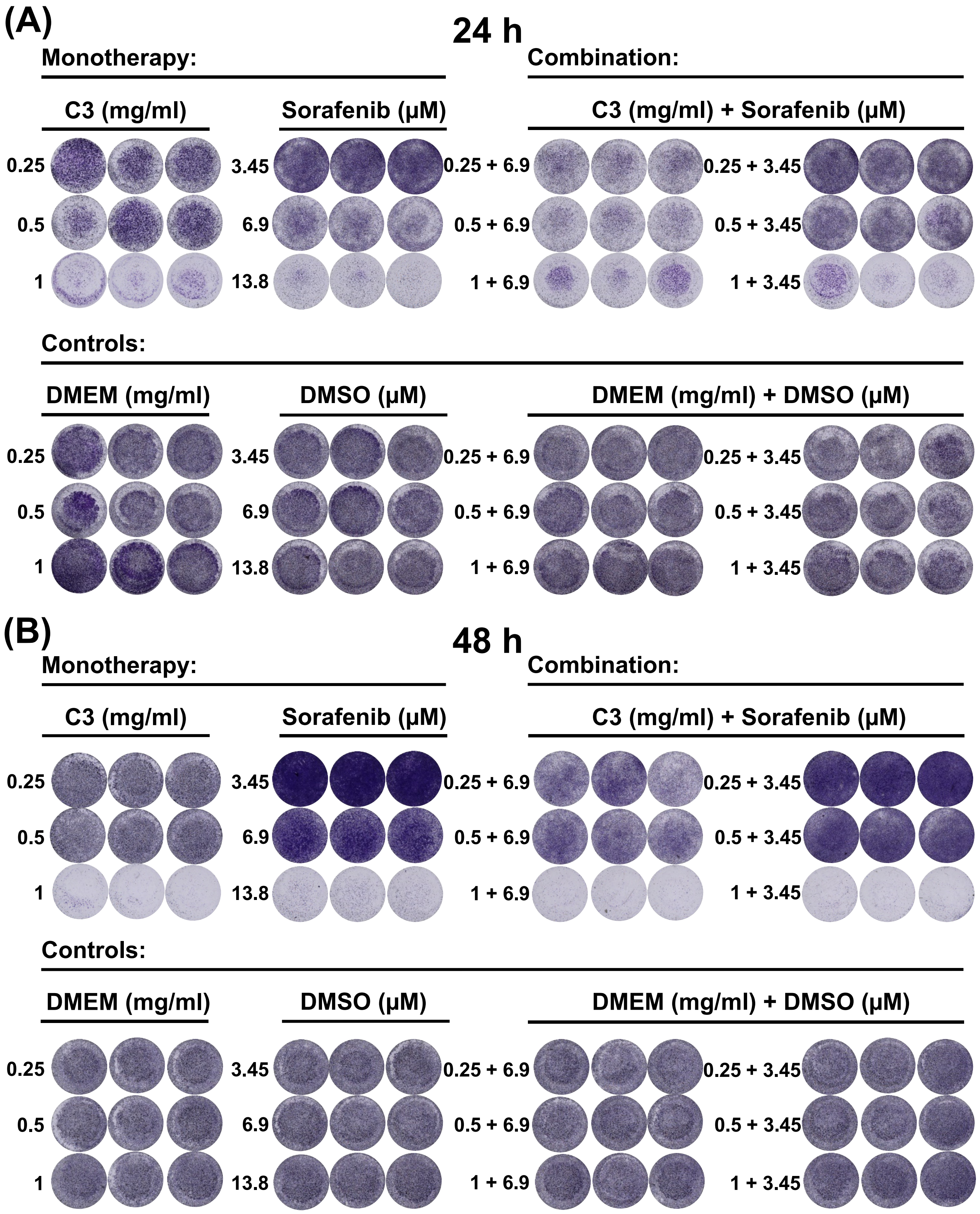

Supplementary Figure 7

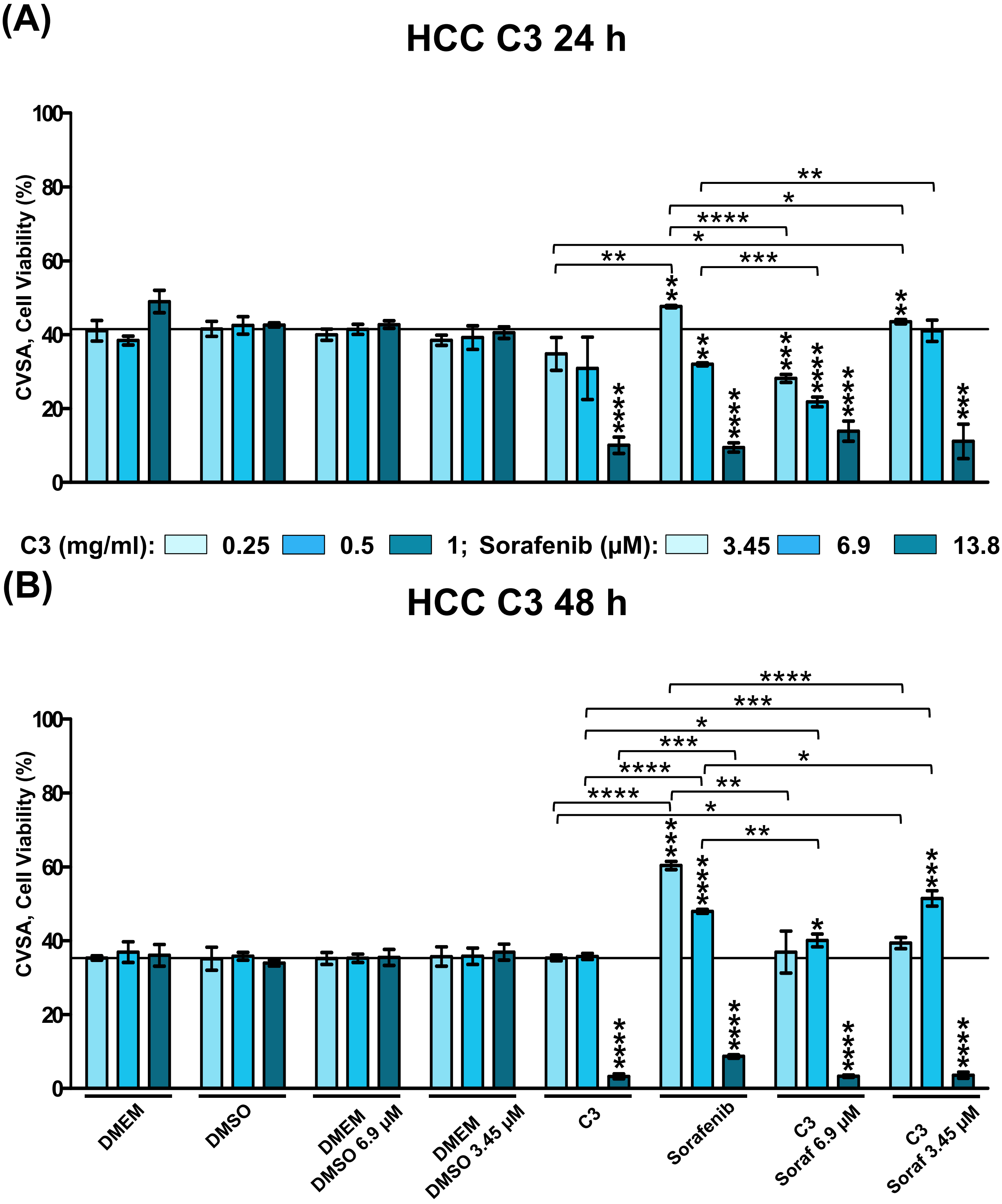

Supplementary Figure 8

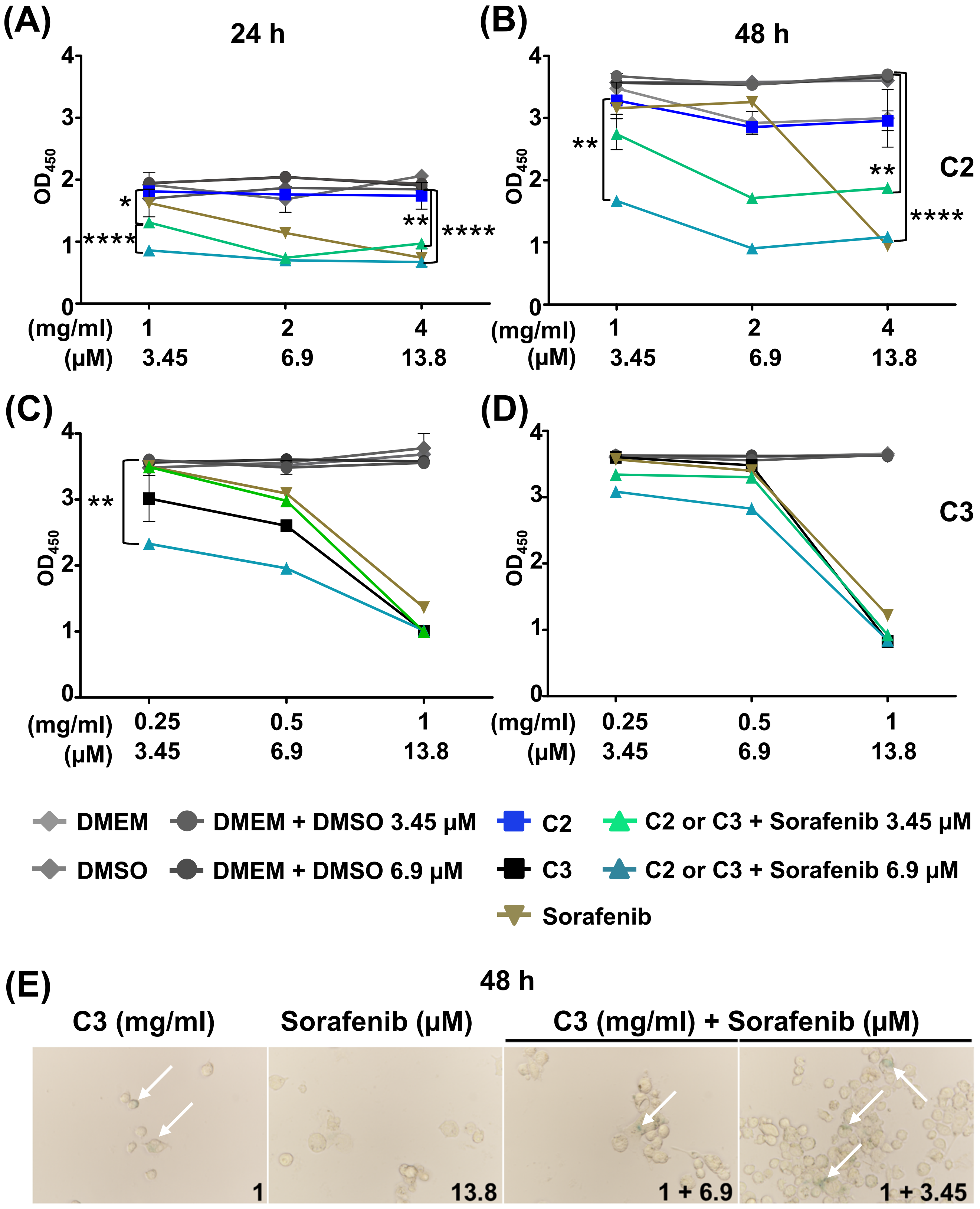

Supplementary Figure 9

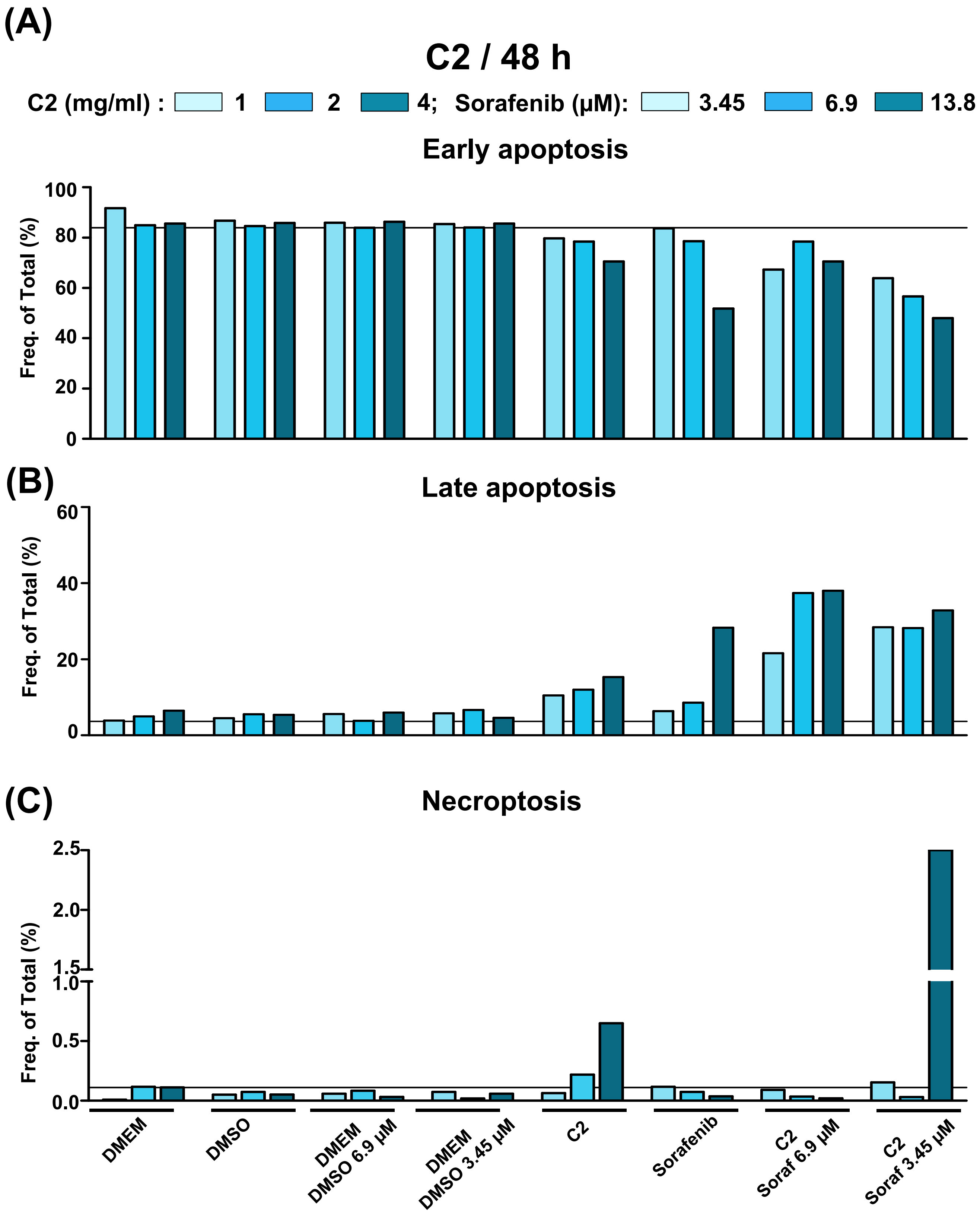

Supplementary Figure 10

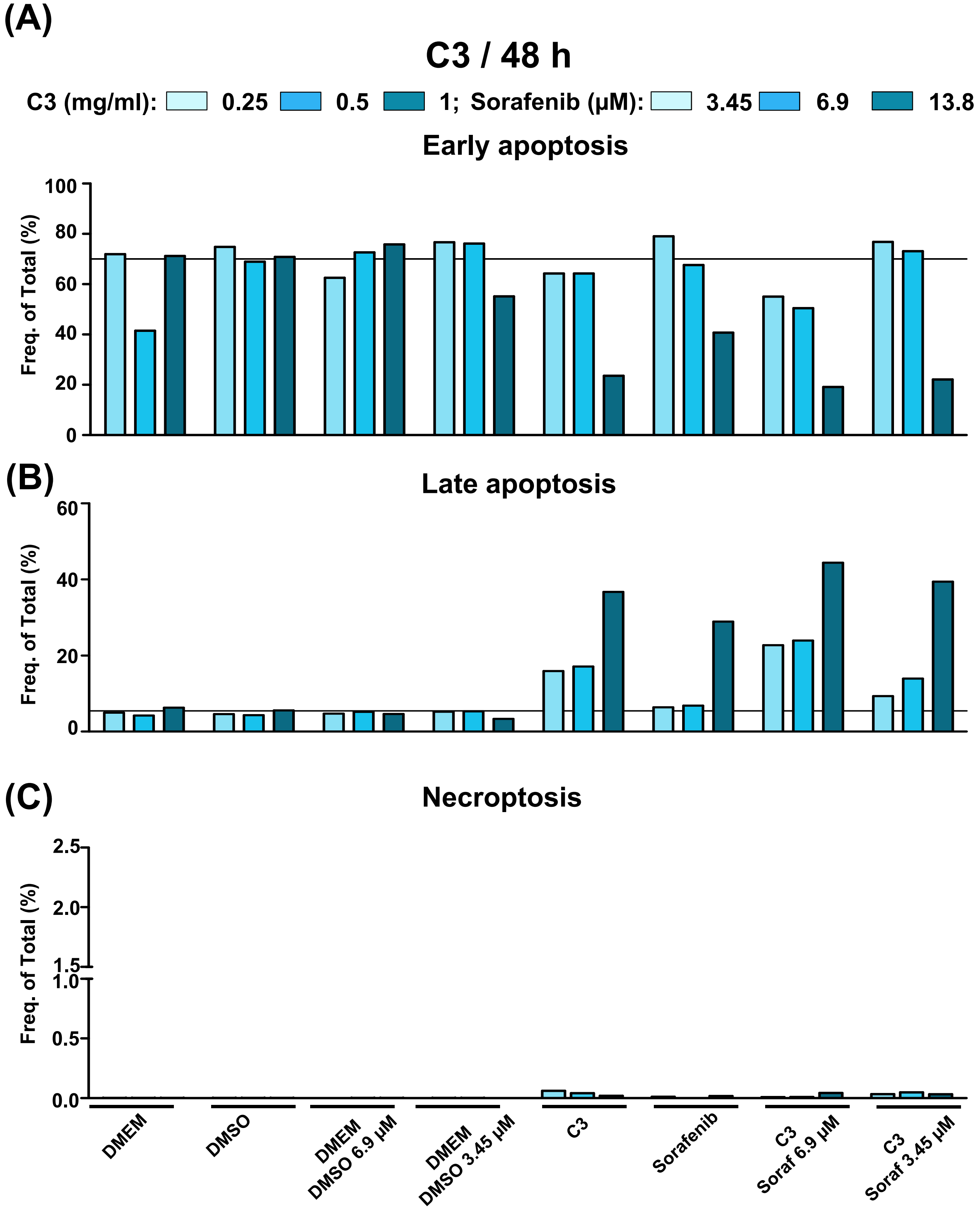

Supplementary Figure 11

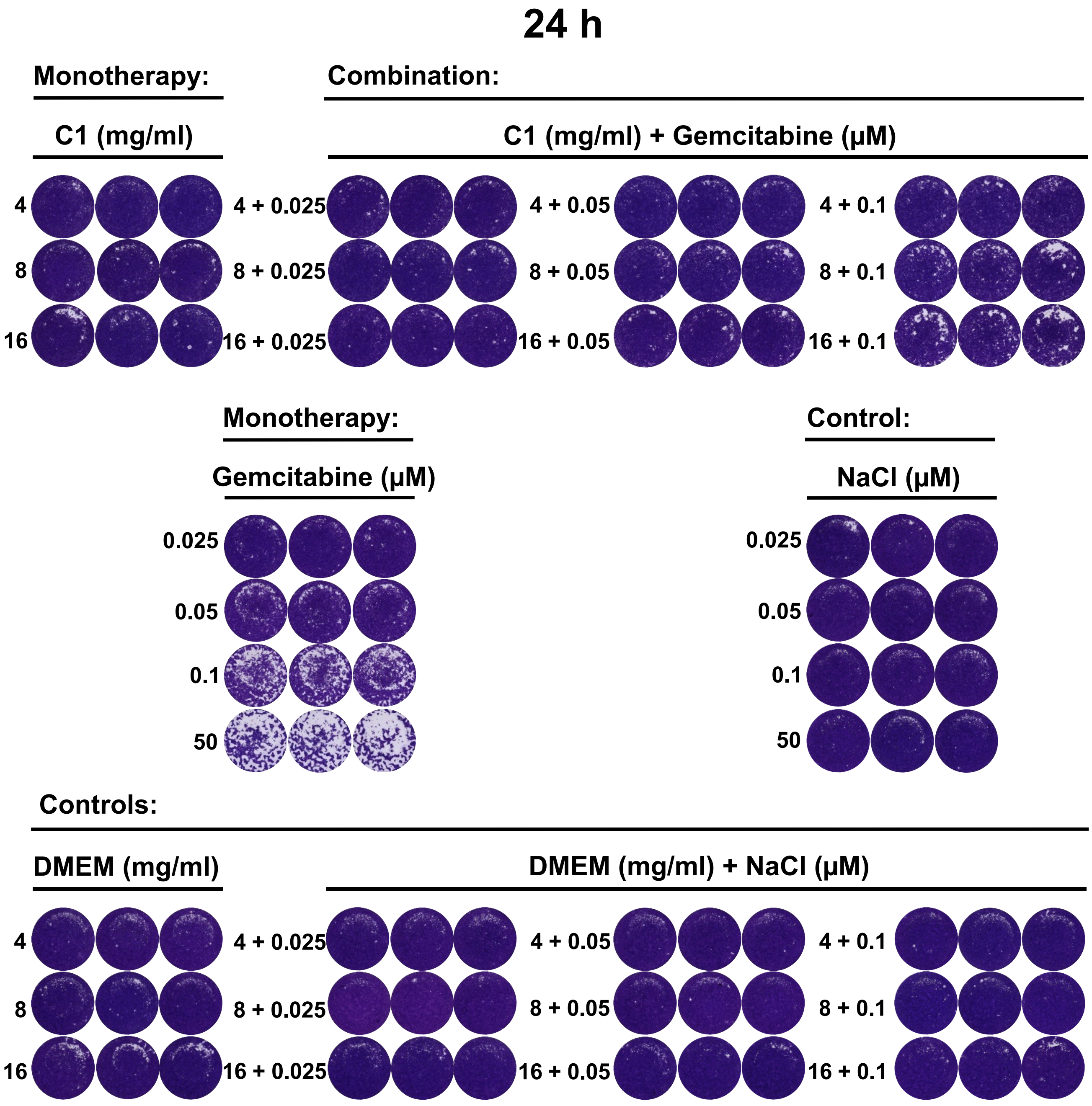

Supplementary Figure 12

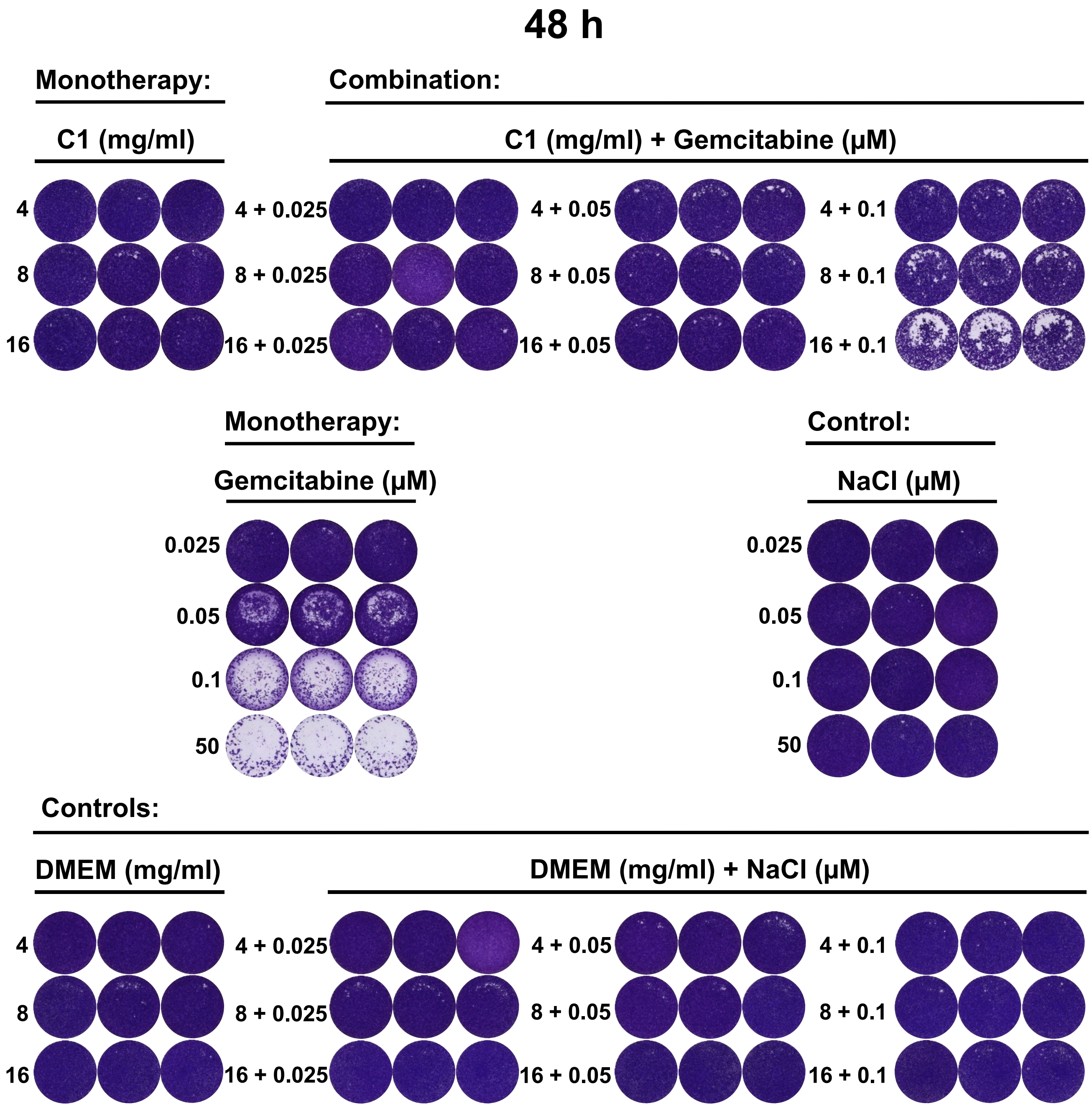

Supplementary Figure 13

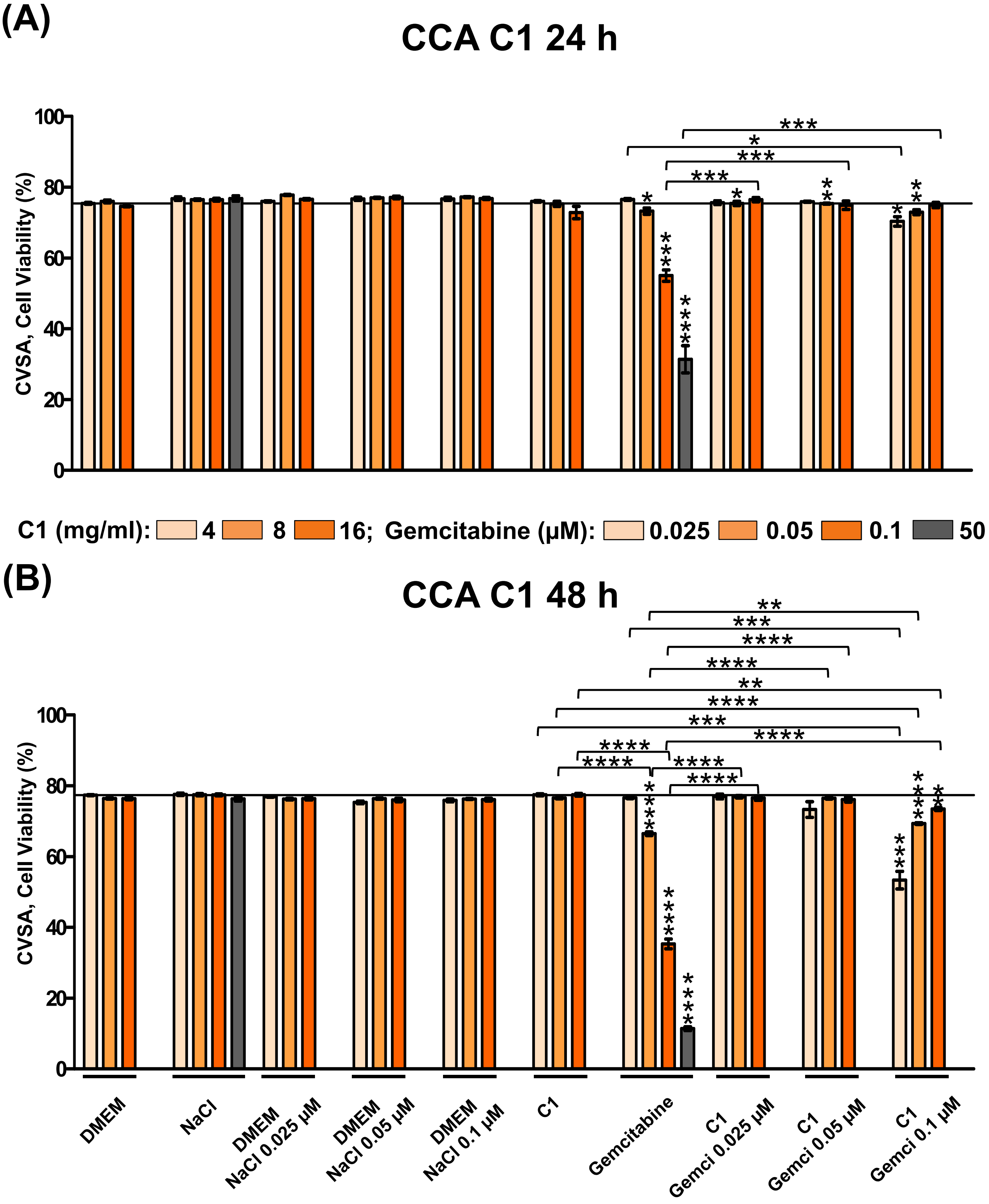

Supplementary Figure 14

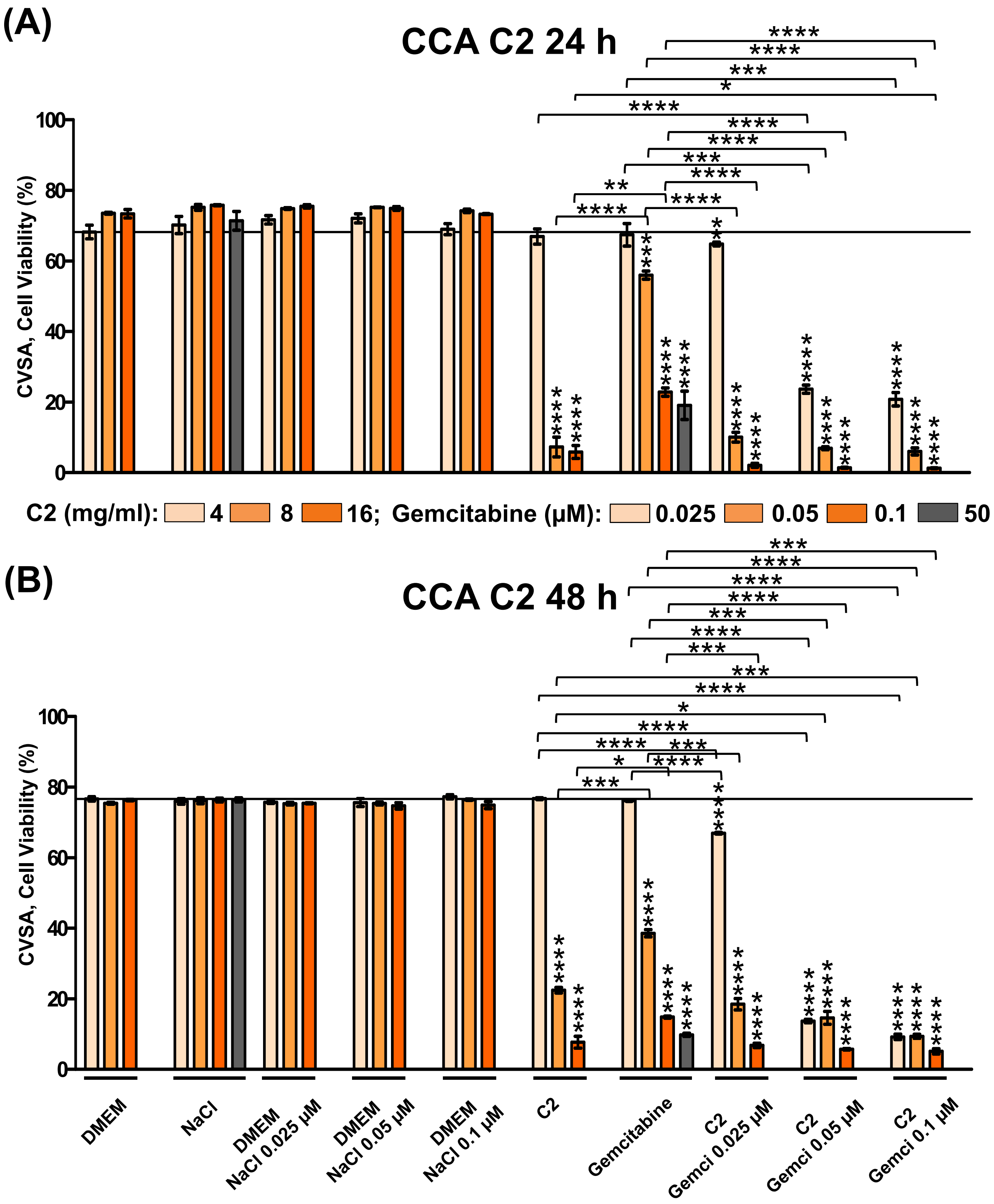

Supplementary Figure 15

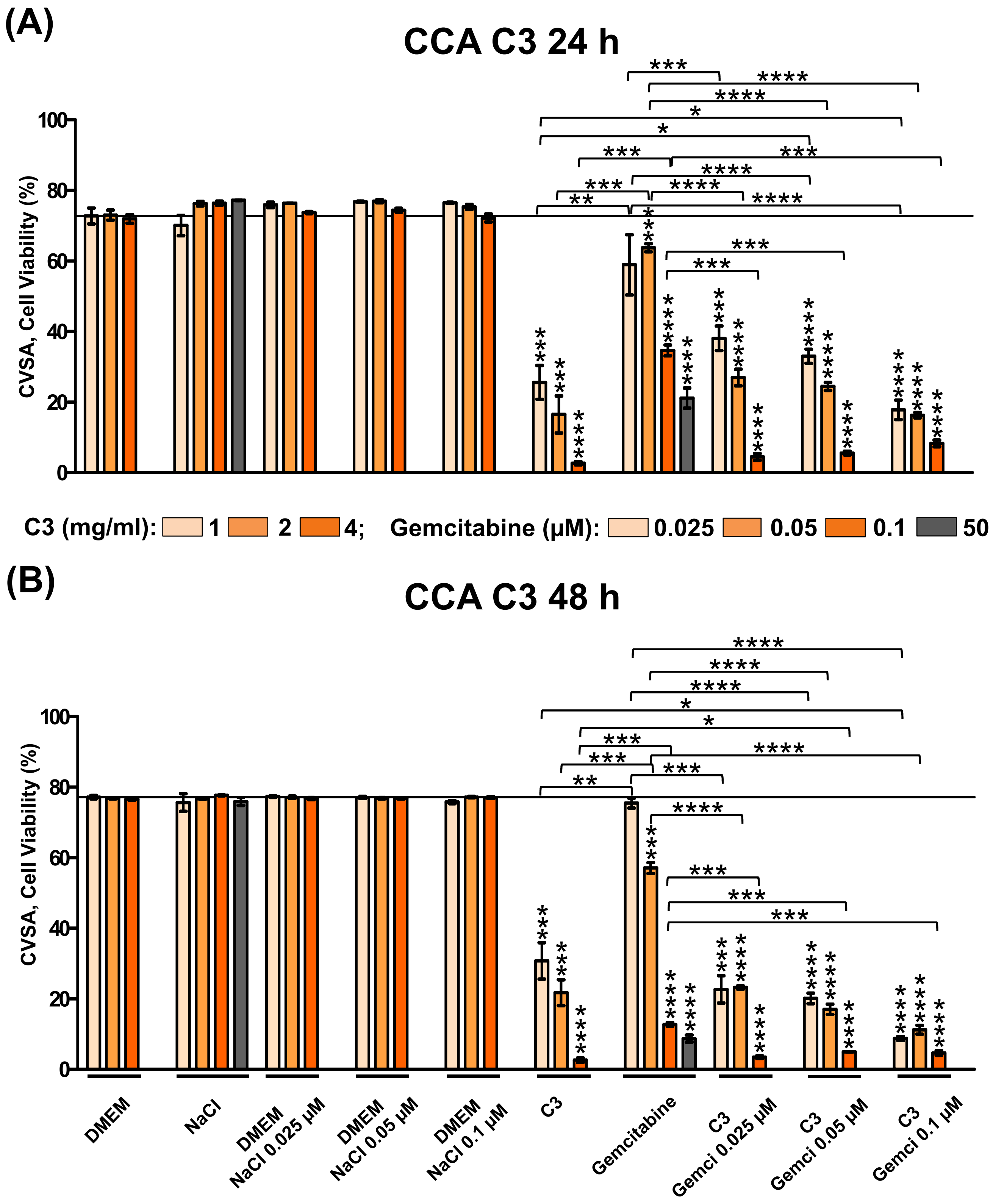

Supplementary Figure 16

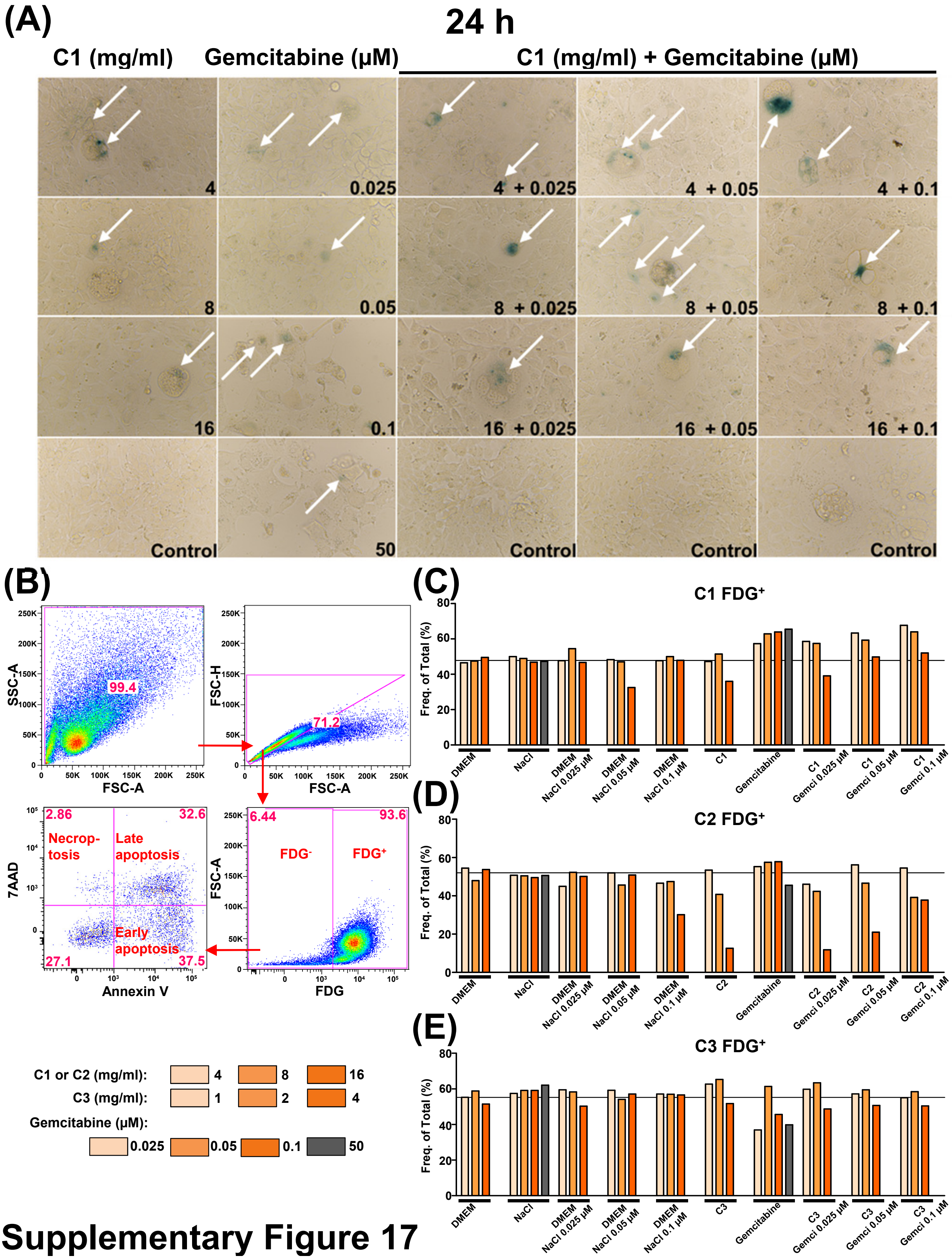

Supplementary Figure 17

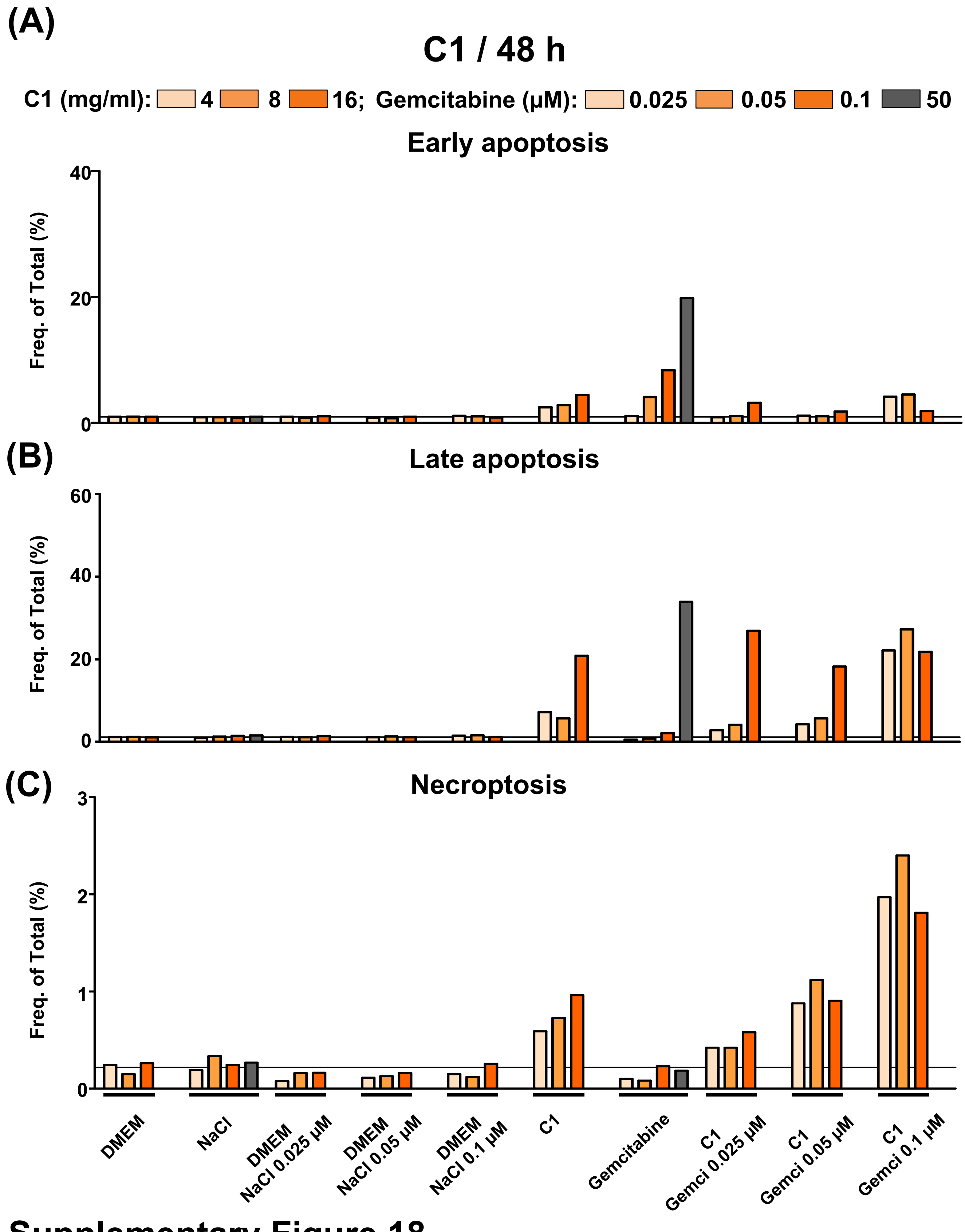

Supplementary Figure 18
